# Safety and efficacy of human ESC-derived corneal endothelial cells for corneal endothelial dysfunction

**DOI:** 10.1186/s13578-023-01145-w

**Published:** 2023-11-06

**Authors:** Juan Yu, Nianye Yu, Yao Tian, Yifan Fang, Bin An, Guihai Feng, Jun Wu, Liu Wang, Jie Hao, Liqiang Wang, Qi Zhou, Wei Li, Yukai Wang, Baoyang Hu

**Affiliations:** 1 Savaid Medical School, University of Chinese Academy of Sciences, Beijing, 100049 China; 2 State Key Laboratory of Stem Cell and Reproductive Biology, Institute of Zoology, Chinese Academy of Sciences, Beijing, 100101 China; 3 Institute of Stem Cell and Regeneration, Chinese Academy of Sciences, Beijing, 100101 China; 4 Beijing Institute for Stem Cell and Regenerative Medicine, Beijing, China; 5 National Stem Cell Resource Center, Chinese Academy of Sciences, 100101, Beijing, China; 6 University of Chinese Academy of Sciences, 100864, Beijing, China; 7 Department of Ophthalmology, The First Center of the PLA General Hospital, Beijing, China

**Keywords:** Human embryonic stem cells (hESCs), Induced corneal endothelial cells (iCECs), Corneal endothelial dysfunction, Cell therapy

## Abstract

**Background:**

Research on human pluripotent stem cells (hPSCs) has shown tremendous progress in cell-based regenerative medicine. Corneal endothelial dysfunction is associated with the loss and degeneration of corneal endothelial cells (CECs), rendering cell replacement a promising therapeutic strategy. However, comprehensive preclinical assessments of hPSC-derived CECs for this cell therapy remain a challenge.

**Results:**

Here we defined an adapted differentiation protocol to generate induced corneal endothelial cells (iCECs) consistently and efficiently from clinical-grade human embryonic stem cells (hESCs) with xeno-free medium and manufactured cryopreserved iCECs. Cells express high levels of typical CECs markers and exhibit transendothelial potential properties in vitro typical of iCECs. After rigorous quality control measures, cells meeting all release criteria were available for in vivo studies. We found that there was no overgrowth or tumorigenicity of grafts in immunodeficient mice. After grafting into rabbit models, the surviving iCECs ameliorated edema and recovered corneal opacity.

**Conclusions:**

Our work provides an efficient approach for generating iCECs and demonstrates the safety and efficacy of iCECs in disease modeling. Therefore, clinical-grade iCECs are a reliable source for future clinical treatment of corneal endothelial dysfunction.

**Supplementary Information:**

The online version contains supplementary material available at 10.1186/s13578-023-01145-w.

## Introduction

The corneal endothelium is a monolayer of polygonal cells forming the innermost layer of the cornea. The pump function of CECs is responsible for regulating stromal dehydration, which plays an important role in maintaining corneal transparency [1–3]. Loss and degeneration of CECs caused by aging, injury or disease leads to stromal edema and visual impairment [4]. Since adult human CECs are incapable of regeneration in vivo [5], endothelial keratopathy caused by corneal endothelial dysfunction has become one of the most serious blinding corneal diseases [6]. The current most effective treatment for corneal endothelial dysfunction is corneal transplantation. It is conservatively estimated that approximately 12.7 million people worldwide need corneal transplantation, and corneal endothelial dysfunction comprises 50% of all corneal transplants performed [7]. Due to the severe shortage of donor corneas, less than 1.5% of patients can receive a transplant, which restricts the widespread use of this therapy [7].

An alternative approach is the transplantation of in vitro expanded primary CECs (pCECs). The results of recent clinical trials showed that cultured pCECs suspensions injected into the anterior chamber survived well and alleviated corneal edema. Most patients exhibited clinical improvement and maintained obvious visual acuity during the 5-year follow-up [8, 9]. However, the limited expansion ability of primary cells makes this treatment highly dependent on corneal donation. Importantly, batch and individual differences in primary cells might bring uncertainty to cell quality control and functional evaluation.

Human pluripotent stem cells (hPSCs) are capable of self-renewal and multilineage differentiation [10–12]; therefore, iCECs derived from hPSCs represent an ideal source for cell replacement therapy. Encouraged by the findings of primary cell transplantation, several differentiation protocols that induce iCECs from hPSCs have been established [13–20] and showed corneal function recovery in animal models of corneal endothelial dysfunction [21–26]. However, challenges remain. There are several barriers to the preparation of hPSC-derived iCECs. First, several hPSC-based iCECs protocols utilize primary cell line coculture or animal-derived components to direct the differentiation of hPSCs into iCECs [13, 15, 17, 18, 23]. To prepare clinical-grade iCECs, xeno-free protocols are preferred. Second, iCECs fate is currently not well characterized. The expression ratio of a single marker is usually used to define iCECs, and comprehensive quality tests, especially in vitro identification of cell function, are lacking. Third, the regulatory criteria of the quality control (QC) check point and iCECs release for manufacturing have not been established.

Here, we developed an efficient xeno-free protocol to generate iCECs using clinical-grade hESC lines and manufactured many cryopreserved iCECs. We also developed a QC system and release criteria for iCECs by testing multiple cell markers and in vitro pump function. Notably, predictive markers expressed in mature iCECs that may correlate with in vitro function were identified. Finally, we demonstrated that the transplantation of iCECs had no toxicity or tumorigenicity and grafts that survived exerted functional recovery in rabbit models of corneal endothelial dysfunction.

## Results

### Generation and in vitro assessment of neural crest cells from hESCs

**Fig. 1:**
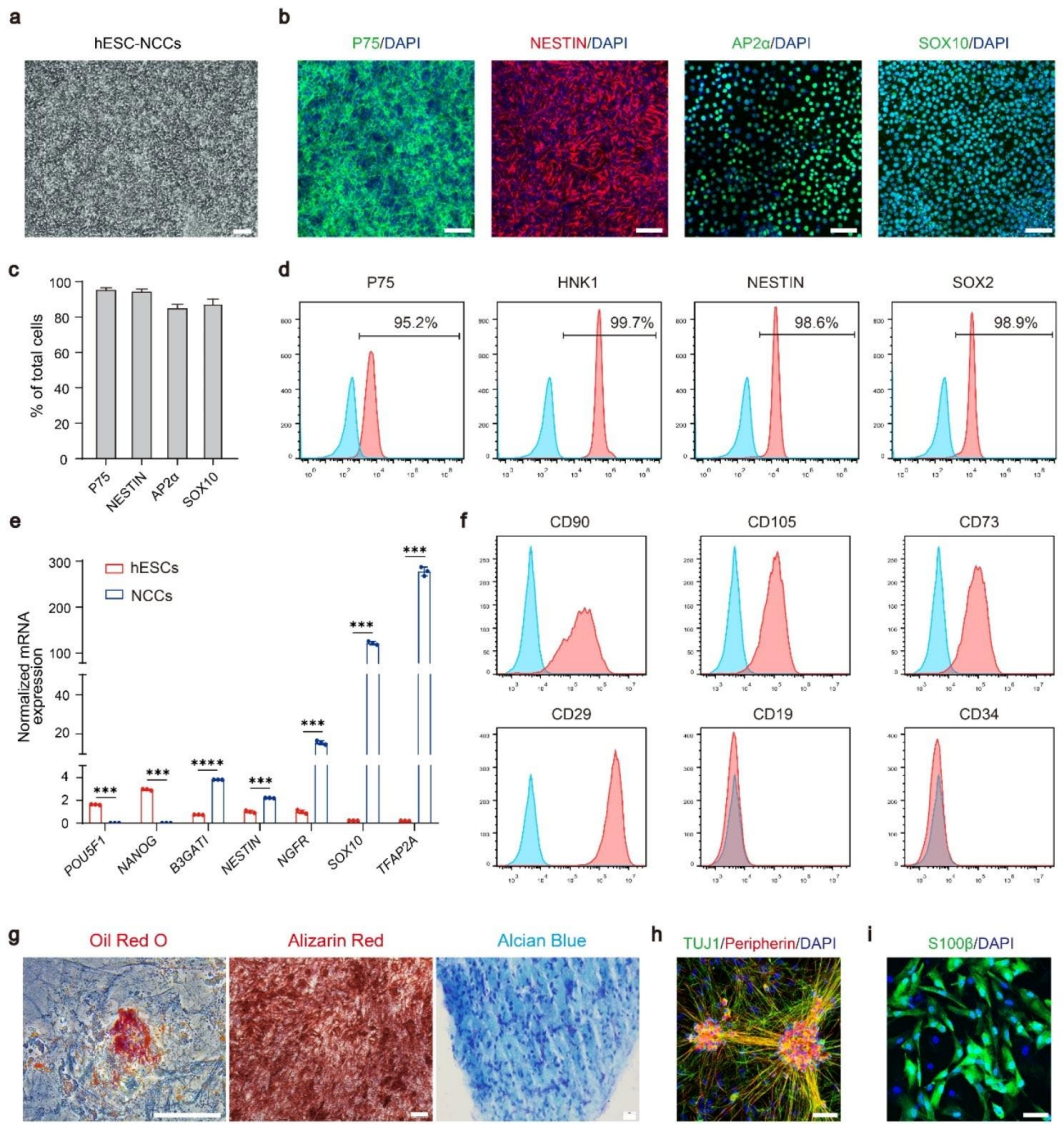
Direct differentiation of hESCs into neural crest cells. **a** hESC-derived NCCs have the typical morphology of neural crest cells. Scale bars: 100 μm. **b** Immunofluorescence staining showing that hESC-derived NCCs expressed P75, NESTIN, AP2α, and SOX10. Nuclei were stained with DAPI. Scale bar: 50 μm. **c** Quantification of the percentage of P75+, NESTIN+, AP2α + and SOX10 + cells at day 12 of differentiation. **d** Flow cytometry analysis of P75, HNK1, NESTIN and SOX2 expression in hESC-derived NCCs. These images are representative of *n =* 3 biological replicates. **e** The expression of pluripotency genes (*POU5F1, NANOG*) and NCCs markers (*B3GATI, NESTIN, NGFR, SOX10, TFAP2A*) in hESCs and hESC-derived NCCs was detected by qPCR. *n =* 3, ^*^p < 0.05, ^**^p < 0.01, ^***^p < 0.001, ^****^p < 0.0001. **f** Flow cytometry analysis of CD90, CD105, CD73, CD29, CD19 and CD34 expression in hESC-NCC-derived mesenchymal stem cells. *n =* 3. **g** hESC-NCCs could be differentiated into mesenchymal derivatives, including Oil Red O-stained adipocytes (red), Alizarin red-stained osteocytes (red), and Alcian blue-stained chondrocytes (blue). Scale bars, 100 μm. **h** Immunofluorescence staining showing that hESC-derived NCCs could be differentiated into peripherin neurons, which expressed TUJ1 and Peripherin. Nuclei were stained with DAPI. Scale bar: 50 μm. **i** S100β + Schwann cells were detected in differentiated hESC-derived NCCs. Nuclei were stained with DAPI. Scale bar: 50 μm

To generate iCECs, we first established a chemically defined differentiation protocol for neural crest cells (NCCs) from hESCs (Fig. S1a). We found that all three hESC lines differentiated into NCCs after 10 days of induction, and the cells expressed the NCCs markers P75, NESTIN, AP2α and SOX10 (Fig. 1a-b). Flow cytometry analysis showed that more than 90% of the cells were positive for P75, HNK1, NESTIN and SOX2 (Fig. 1c). We also confirmed that NCCs can be efficiently generated from two other hESC lines (Fig. S1b-c). qPCR analysis further showed that pluripotency markers, such as *OCT4 (POU5F1)* and *NONOG*, were significantly downregulated, while the expression levels of NCC-specific genes, including *B3GAT1*, *NES*, *NGFR*, *SOX10* and *TFAP2A*, were significantly increased (Fig. 1d).

Given that NCCs are a multipotent cell population that is capable of forming a wide diversity of cell types, including peripheral neurons, Schwann cells and mesenchymal stem cells (MSCs), we next sought to investigate the differentiation potential of hESC-derived NCCs in vitro. As expected, after culture with 10% fetal bovine serum (FBS) medium for 3 weeks, NCCs were differentiated into MSCs, as evidenced by the expression of the MSCs markers CD90, CD105, CD73 and CD29 and the absence of CD19 and CD34 (Fig. 1e). In addition, these MSCs possess adipogenic, osteogenic and chondrogenic potential (Fig. 1f). To assess the neural differentiation potential, hESC-NCCs were induced with media containing BDNF, GDNF, NT3 and dbcAMP. After 21 days, most of the cells co-expressed Peripherin and TUJ1, indicative of peripheral neuron identity. Furthermore, hESC-NCCs could differentiate into S100β + Schwann cells (Fig. 1g). These data suggested successful generation of highly purified NCCs from hESCs.

### Highly efficient differentiation of hESC-NCCs into iCECs

**Fig. 2:**
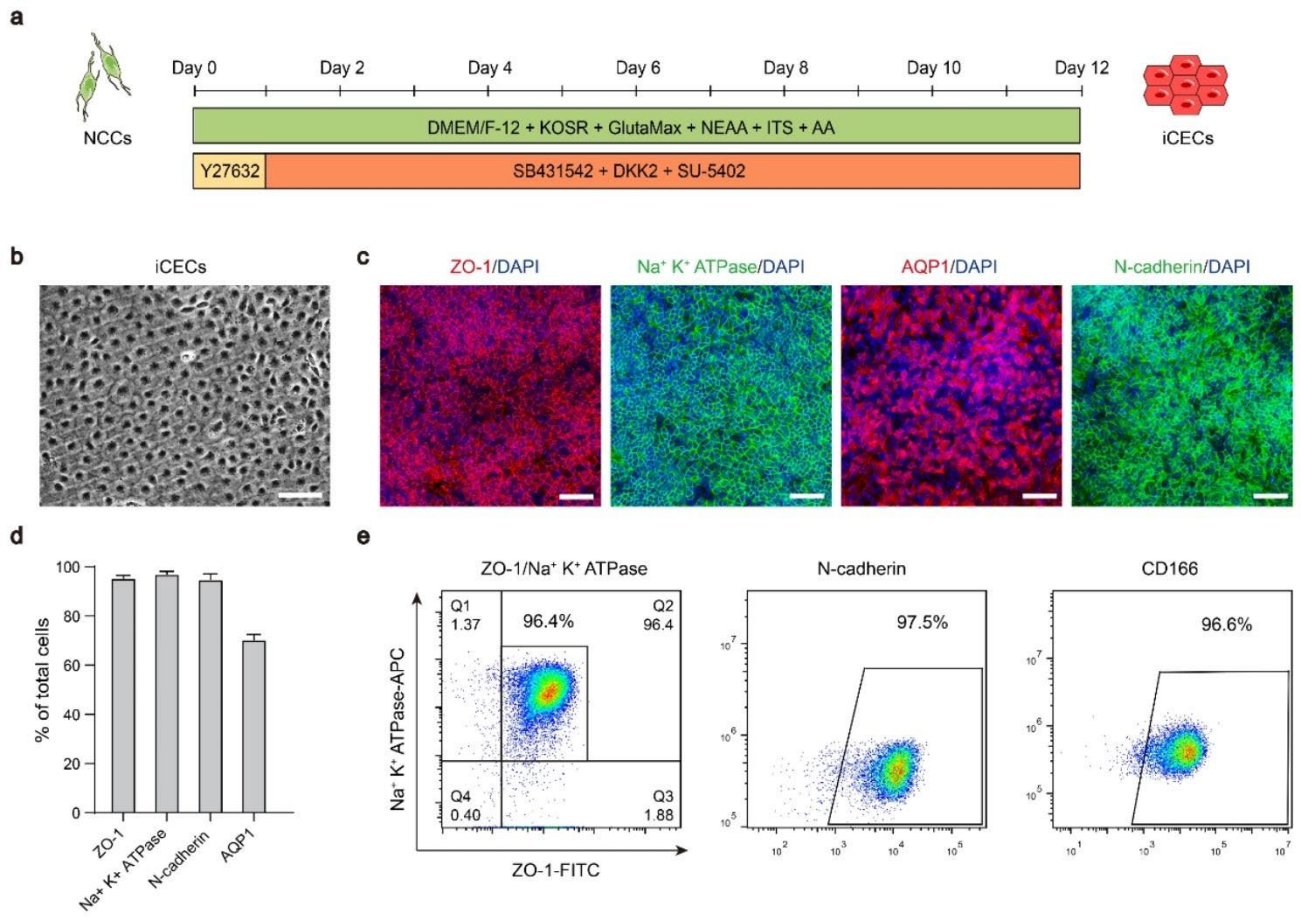
Differentiation of hESC-derived NCCs into corneal endothelial cells. **a** Schematic illustration of the differentiation conditions used to generate corneal endothelial cells from NCCs. **b** The hexagonal morphology of iCECs differentiated from hESC-derived NCCs. Scale bar: 50 μm. **c** Immunofluorescence staining showing that iCECs expressed ZO-1, Na^+^ K^+^ ATPase, AQP1 and N-cadherin. Nuclei were stained with DAPI. Scale bars: 50 μm. **d** Quantification of the percentage of ZO-1+, Na^+^ K^+^ ATPase+, AQP1 + and N-cadherin + cells at day 24 of differentiation. **e** Flow cytometry analysis of ZO-1/Na^+^ K^+^ ATPase, N-cadherin and CD166 expression in iCECs. *n =* 3

To develop a xeno-free iCECs differentiation protocol, we used medium without any animal-derived components and tested numerous combinations of small molecules and factors related to the pivotal pathways of eye development involving TGFβ, WNT and FGFR [27, 28]. We first assessed the efficiency of inducing iCECs using the TGFβ inhibitor SB431542 and the WNT inhibitor DKK2 (SD). Treatment with SD resulted in high expression of ZO-1 by day 12 and a complete lack of Na^+^ K^+^ ATPase expression (Fig. S2a). Fibroblast growth factor 2 (bFGF) has been reported to stimulate endothelial-mesenchymal transition of iCECs during in vitro culture [29, 30]. In subsequent optimization, we found that NCCs failed to produce ZO-1 + or Na^+^ K^+^ ATPase + iCECs after treatment with SD medium supplemented with bFGF (SDB) for 12 days. However, ZO-1 and Na^+^ K^+^ ATPase were robustly induced in SDU (SD medium supplemented with FGFR inhibitor SU-5402)-treated cultures (Fig. S2a), suggesting that inhibition of WNT and TGFβ signaling in combination with inhibition of the FGFR pathway can efficiently convert NCCs to iCECs (Fig. 2a).

After induction for 12 days, the cells exhibited polygonal morphology with tight junctions (Fig. 2b). Immunofluorescence staining revealed that CEC-specific markers, including tight junction protein 1 (ZO-1), Na^+^- and K^+^-dependent adenosine triphosphatase (Na^+^ K^+^ ATPase), N-cadherin, AQP1 and CD166, were highly expressed (Fig. 2c-d). We also established a fluorescence-activated cell sorting (FACS) assay system to enable the quantification and enrichment of differentiated iCECs (Fig. 2e). The robustness of the protocol across cell lines was confirmed by repeated experiments using two additional hESC lines (Fig. S2b).

### Molecular characterization and in vitro functional assay of iCECs during differentiation

**Fig. 3:**
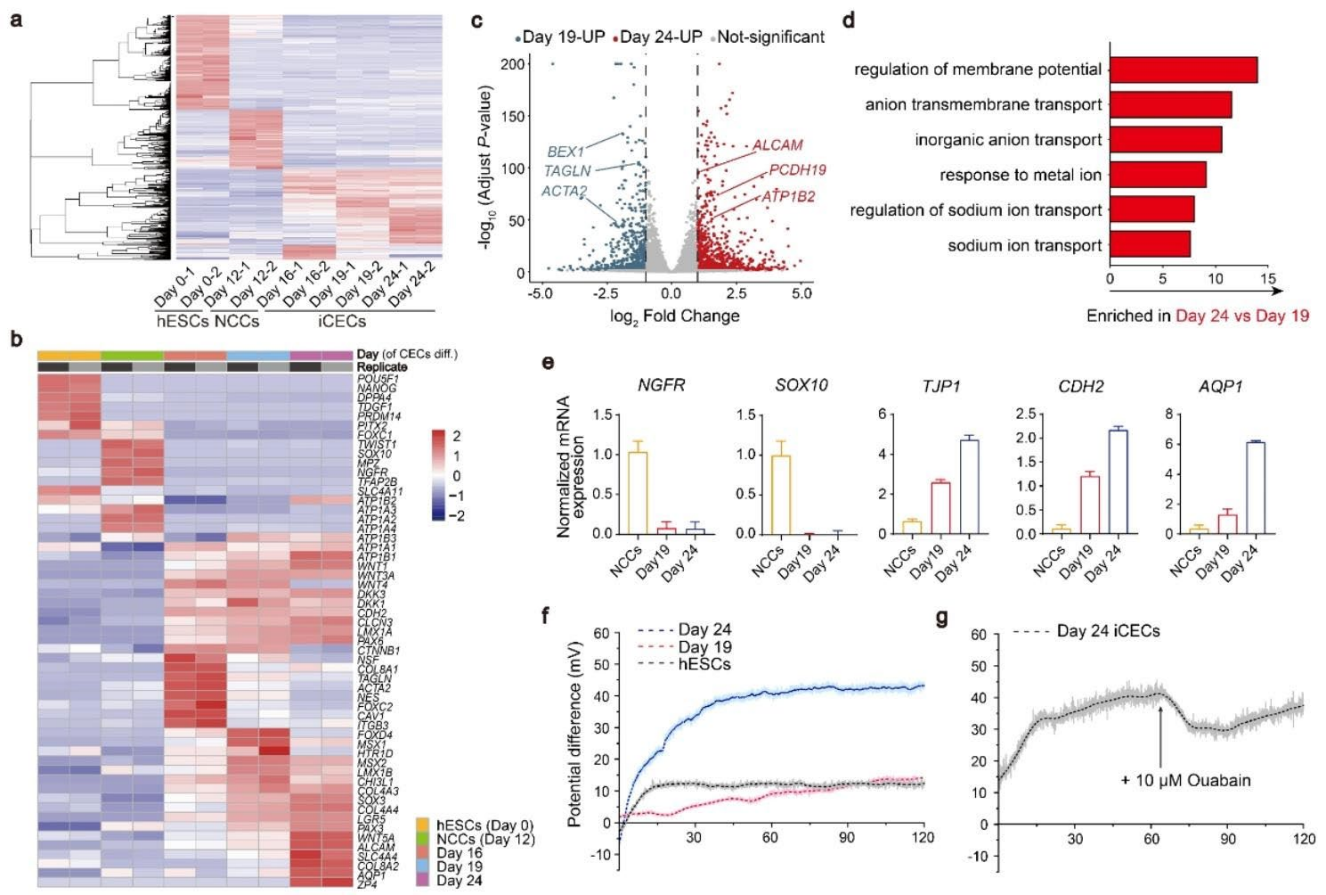
In vitro maturation and functional characteristics of iCECs. **a** Heatmap of RNA expression at different time points in differentiated iCEC. There were 2 independent biological replicates in each sample. **b** Heatmap of selected gene sets from RNA-Seq at different time points of iCECs differentiation. The unit of the color in each gene is log (FPKM + 1). Blue indicates downregulated transcripts, and red indicates upregulated transcripts. **c** Volcano plot of differentially expressed genes between day 19 and day 24 of iCECs differentiation. Blue indicates upregulated transcripts on day 19, and red indicates upregulated transcripts on day 24. **d** GO analysis from RNA-Seq within day 19 versus day 24 iCECs differentiated cells. **e** qPCR of CECs markers during iCECs differentiation of hESC-derived NCCs. **f** The potential differences in hESCs, day 19 and day 24 of differentiated iCECs. The image presents the mean value of 10 times per second. **g** Changes in the potential difference before and after adding 10 µM ouabain to day 24 iCECs. Total time is 120 min (**f, g**)

We next performed RNA sequencing (RNA-seq) to identify the transcriptional expression signatures of cells at different stages from hESCs. Globally, gene expression analysis revealed the progressive differentiation and maturation of hESCs into iCECs by day 24 of differentiation (Fig. 3a). Principal component analysis (PCA) and cluster dendrogram both supported the marked transcriptional changes in the time course of differentiation and demonstrated similar transcriptional profiles of three iCECs groups (days 16, 19 and 24) (Fig. S3a-b). Analysis of pluripotent and differentiated genes confirmed that all groups of iCECs started to express the barrier-associated gene *CDH2 (N-cadherin)* and membrane transport genes *ATP1A1* and *CLCN3*, which were distinct from those of hESCs and NCCs, showing a cell fate transition toward corneal endothelial identities. Moreover, to better define the differentiation of iCECs from hESCs as well as NCCs, gene set enrichment analysis (GSEA) showed that the significantly upregulated gene sets in the pCECs compared with hESCs as well as NCCs, were enriched in the high expression region of iCECs. Conversely, the significantly downregulated gene sets in the pCECs were enriched in the low expression region of iCECs (Fig. S3c-f). The sequencing date of pCECs was from the previous study [15]. These comprehensive results provided valuable insights into the transcriptional differences among pCECs with hESCs as well as NCCs, highlighting the similar gene expression patterns of iCECs with pCECs. Notably, iCECs only at day 24 highly expressed the known CECs markers *COL8A2*, *AQP1*, *ALCAM*, *ZP4* and *SLC4A4*, confirming an authentic CECs phenotype, while other groups displayed lower or no expression of these genes. We also found that there was almost no expression of *SLC4A11* in day 24 cells (FPKM < 1) (Fig. 3b). A recent study showed that CEC precursors didn’t express *SLC4A11* until 7 days after transplantation [26]. Given that *SLC4A11* is a marker of mature iCECs, our day 24 cells may need further maturation in vivo.

Differentially expressed genes (DEGs) analysis comparing the two stages (day 19 and day 24) showed a total of 8039 genes that were differentially expressed (*p* value adjusted < 0.05). The upregulated genes on day 24 (*ALCAM*, *PCDH19*, *ATP1B2*) were associated with cell adhesion and migration, osmotic regulation and ion transportation. However, the downregulated genes (*BEX1*, *TAGLN*, *ATCA2*) were associated with the formation of neural cells and smooth muscle, which are derivatives of NCCs (Fig. 3c). Gene Ontology (GO) analysis revealed that genes enriched at day 24 relative to day 19 were largely associated with regulation of membrane potential, anion membrane transport and inorganic anion transport, consistent with the specific characteristic of mature iCECs that had been reported in previous work [15], which may be the functional features that distinguish early and mature iCECs (Fig. 3d). qPCR analysis confirmed the dramatic decrease in NCCs markers (*NGFR* and *SOX10*) and a time-dependent increase in CECs markers (*TJP1*, *CDH2* and *AQP1*) (Fig. 3e).

Given that *TJP1 (ZO-1)*, *CDH2 (N-cadherin)*, *ATP1A1 (Na*^*+*^*K*^*+*^*ATPase)* and *AQP1* play critical roles in the function of CECs, we hypothesize that a prolonged maturation culture might be needed for functional acquisition of iCECs. To demonstrate the functional capacity of iCECs, we performed transendothelial electrical potential difference recording of day 19 and day 24 cells using a Transwell system as described previously [31]. This transendothelial potential is dependent on continuous tight junctions and the activity of Na^+^ K^+^ ATPase, which is of fundamental importance for the osmotic activity of iCECs. Compared with the hESCs control, the time course of the potential difference of day 24 cells showed a rapid increase after recording. The plateau value was approximately 42 mV within 45 min, while the potential difference of day 19 cells only rose to 14 mV slowly until 120 min (Fig. 3f). Notably, the potential difference can be partially blocked by ouabain, a Na^+^ K^+^ ATPase inhibitor that is used to treat heart failure and arrhythmias [32] (Fig. 3g). These data indicate that day 24 cells subjected to our differentiation protocol acquire the key functionality of iCECs.

### Generation of a clinically available off-the-shelf iCEC products

**Fig. 4:**
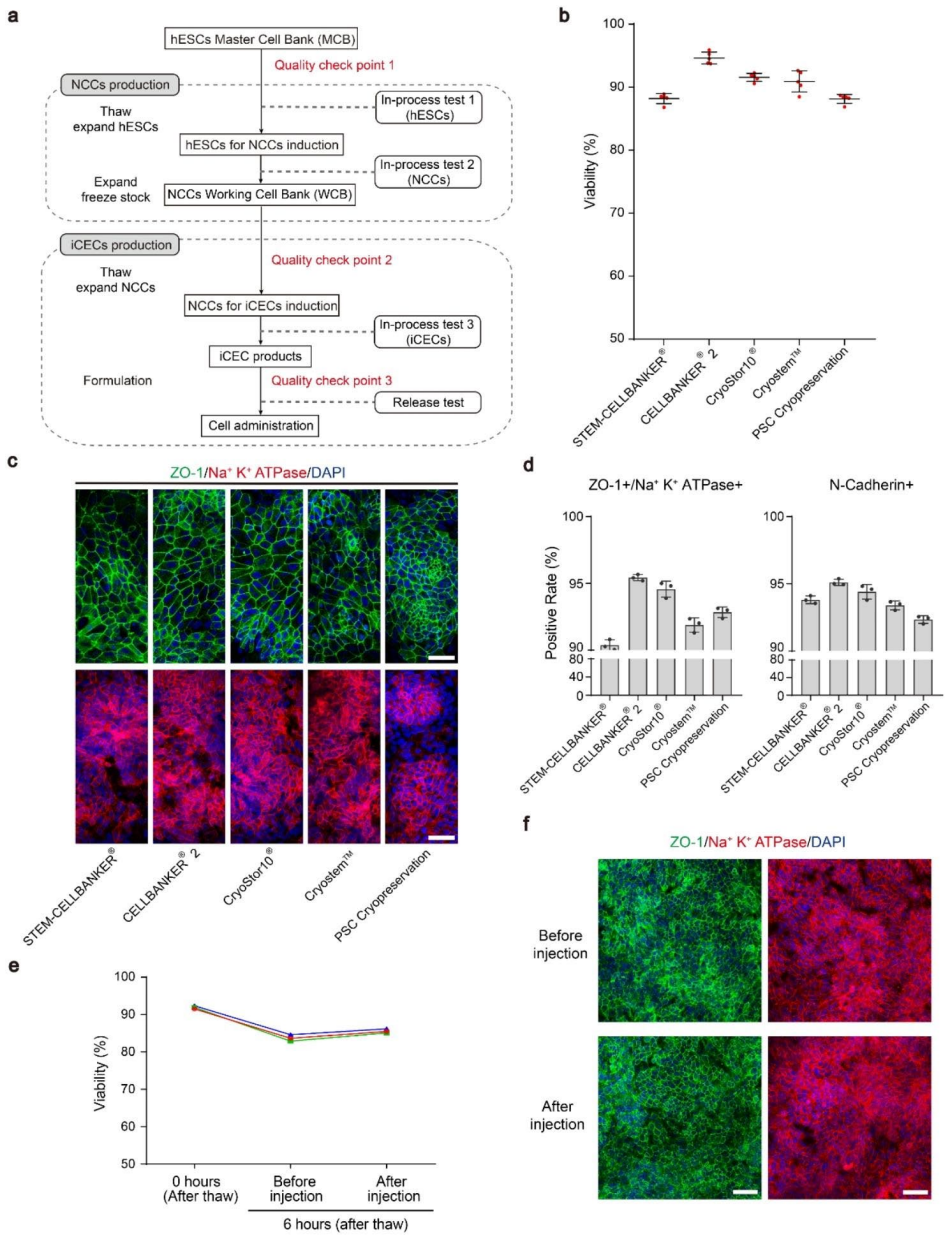
Characterization of clinically available iCEC products. **a** Scheme of the cell production process for clinical use. **b** Viability of cryopreserved iCEC products postthawing in several commercial cryopreservation reagents. Cell viability was measured by the AOPI system. **c** Immunofluorescence staining showing that the iCEC products expressed ZO-1 and Na^+^ K^+^ ATPase. Nuclei were stained with DAPI. Cells were analyzed after 5 days of further iCECs differentiation postthawing the day 24 iCEC products. The reagents used for each cell freezing are shown at the bottom. Scale bars: 50 μm. **d** Quantification of the positive rate of ZO-1/Na^+^ K^+^ ATPase and N-cadherin cells in cryopreserved iCECs after flow cytometry analysis. **e** The viability of postthawing iCEC products was measured at 0 h and in cells placed on ice for 6 h before and after simulated injection. These lines represent 3 independent experiments. **f** Immunofluorescence staining of ZO-1 and Na^+^ K^+^ ATPase for iCEC products, which were cultured for 5 days from **e**. Cells were on ice for 6 h prior to stimulation injection. Nuclei were stained with DAPI. Scale bars: 50 μm

The workflow diagram for the overall cell manufacturing process is shown in Fig. 4a. Briefly, the hESCs master cell bank (MCB) was established using the CB0019 cell line (passage 30) under current good manufacturing practice (cGMP) conditions. For NCCs production, one frozen vial from MCB was thawed and expanded for 3 passages prior to differentiation. After differentiation for 12 days, the NCCs working cell bank (WCB) was established. For iCECs production, one of the vials from WCB was thawed and directly differentiated into iCECs. The final products were harvested and cryopreserved at differentiation day 24. In the process of cell manufacturing, quality control check points were placed to ensure the quality of the MCB and WCB intermediate products, and the final products had to pass the release test before it could be used (Fig. 4a and Table S1).

To ensure the long-term stability of the cryopreserved iCEC products in a ready-to-use format, we first compared various commercial cryopreservation media and determined the postthaw viability of the iCEC products after 6 months. CELLBANKER 2 yielded the highest viability after thawing, which ranged from 93.73 to 95.91% (average 94.64% ± 0.94% n = 5 vials/lot) (Fig. 4b). Immunofluorescence staining showed that cells in the CELLBANKER 2 group exhibited robust ZO-1 and Na^+^ K^+^ ATPase expression with the expected CECs morphology (Fig. 4c). Flow cytometry analysis confirmed the high percentages of ZO-1, Na^+^ K^+^ ATPase double-positive (95.4%) and N-cadherin (95.1%) positive cells (Fig. 4d). Moreover, cells can maintain a viability of more than 80% for 6 h post-thawing in injection solution at 4℃. Importantly, according to our cell transplantation protocol, iCECs can be easily aspirated with a 30G insulin needle (0.3 mm inner diameter) and yield high postinjection viability (85.1%), allowing the transport of iCEC products to the surgical site (Fig. 4e). There was no significant difference in the expression of ZO-1 and Na^+^ K^+^ ATPase before and after injection (Fig. 4f). Overall, this off-the-shelf platform enables a rigorously quality controlled batch of cells for clinical use.

### Residual hESCs and teratoma formation assays of iCECs

**Fig. 5:**
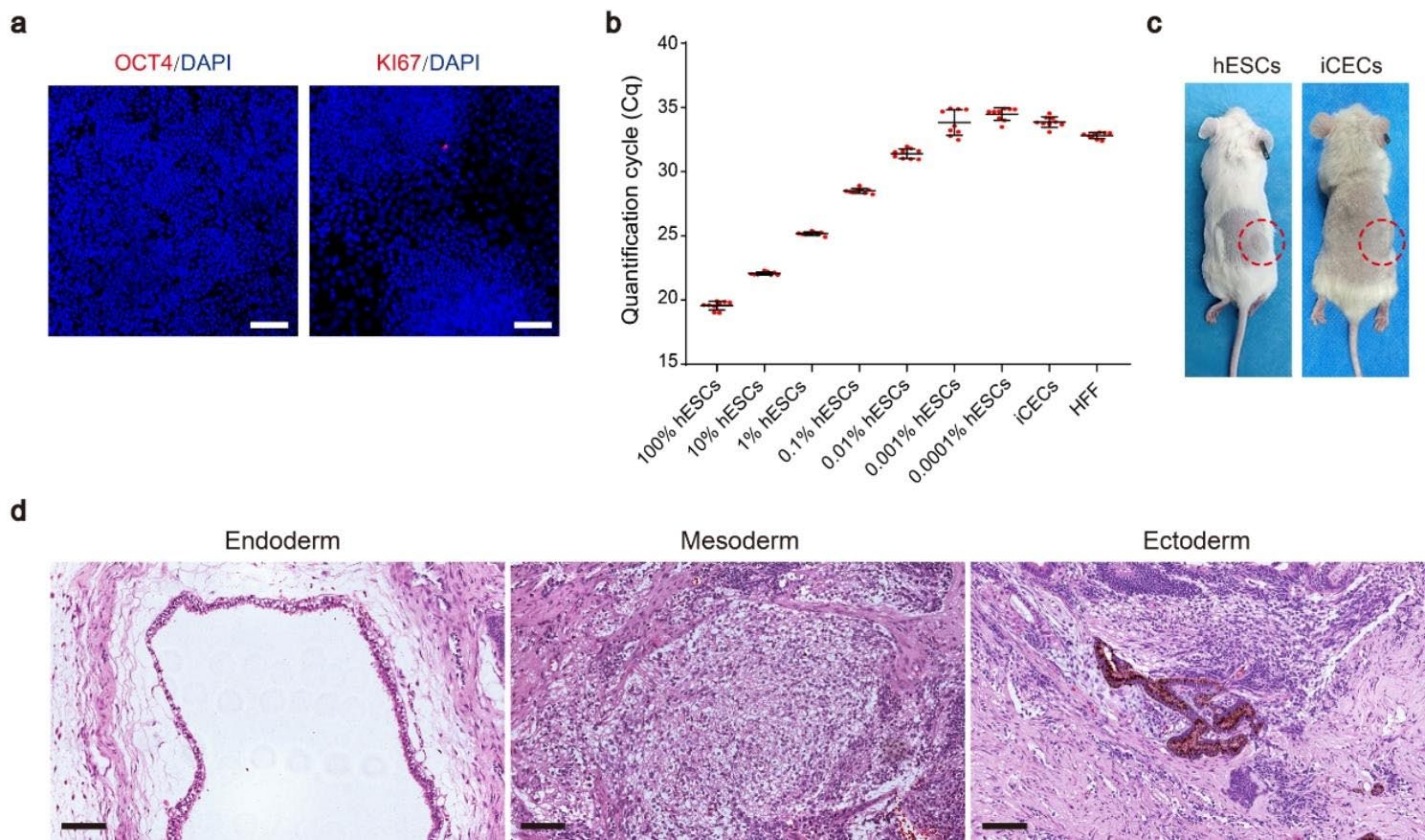
Results of the in vivo safety study. **a** Immunofluorescence staining of OCT4 and KI67 for iCEC products. Nuclei were stained with DAPI. Scale bars: 50 μm. **b** qPCR analysis of *POU5F1* in iCEC products spiked with different concentrations of hESCs. The data are represented as quantification cycle values (Cq) (mean ± SD, *n =* 9) and normalized to *GAPDH*. **c** Tumorigenicity study of hESCs (number of animals = 10) and iCEC products (number of animals = 20). **d** Representative images of hematoxylin and eosin staining showing the endoderm (respiratory epithelium), mesoderm (cartilage) and ectoderm (pigmented retinal epithelium) from a single teratoma in the subcutaneous space of NOG mice injected with hESC lines (CB0019). Scale bars: 100 μm

Teratoma formation caused by undifferentiated pluripotent cells is considered to be a major concern for the application of hPSC-derived cells. Our immunofluorescence staining showed that no residual OCT4 + or KI67 + cells remained in the final products (Fig. 5a). To further examine the existence of residual hESCs, a qPCR assay for *POU5F1 (OCT3/4)* was performed. Samples containing 100% hESCs (positive control), human foreskin fibroblasts (HFF, negative control) and iCECs spiked with 0.00001–10% hESCs were used to determine the limit of detection. The results reveal that the detection limit for this assay is approximately 0.001%, which is consistent with a recently published article [33]. Importantly, the expression level of *POU5F1* in the HFF negative control was significantly higher than that in day 24 iCECs (^**^p < 0.01) (Fig. 5b), suggesting that *POU5F1* mRNA was undetectable in the final products.

To examine the long-term safety of iCECs in vivo, we transplanted day 24 cells (1 × 10^6^ cells per mouse) into the subcutaneous space of NOG SCID mice, which is the same dose used in the subsequent efficacy test. The hESC line CB0019 (1 × 10^6^ cells per mouse) was used as a positive control. As expected, hESCs gave rise to teratomas in all 10 mice at approximately 4–10 weeks. Histological analysis showed typical teratomas containing cells in 3 germ layers. In contrast, no teratoma formation was observed for over 6 months in the iCECs group (n = 20) (Fig. 5c-d). Moreover, no unexpected death was observed in this group. These results suggest that day 24 iCECs do not contain undifferentiated hESCs or teratomas.

In addition, to determine the survival and in vivo biodistribution of transplanted iCECs, we injected Luciferase-labeled iCECs into the anterior chamber of rabbits with corneal endothelial dysfunction. The bioluminescence intensity of graft-surviving rabbits decreased from day 14 to day 28, indicating that the number of transplanted iCECs was decreasing. However, there was no bioluminescence signal in the vehicle group (Fig. S4a-b). Meanwhile, no bioluminescence signal was observed in other parts of the body (Fig. S4a). These results indicate that the transplanted iCECs were distributed in the eyes without overgrowth.

### In vivo survival and efficacy study of iCECs in rabbit models

To evaluate the in vivo potential of differentiated iCECs, we transplanted iCECs into unilaterally lesioned rabbit models. All studies were performed under good laboratory manufacturing practice (GLP) conditions. First, we mechanically scraped unilateral corneal endothelium from Descemet’s membrane of rabbits as described previously to construct a rabbit corneal endothelial dysfunction model [34]. To confirm that rabbit cornea endothelium was completely scrapped off after modeling, we performed alizarin red staining of the corneas immediately after surgery. The results showed that there were no residual CECs in rabbit models, while CECs of WT group showed obvious hexagonal cell morphology (Fig. S5a). Moreover, confocal microscopy examination (HRT3) examination of vehicle group rabbits (*n* = 6) showed that no CECs were observed 1 day after surgery (Fig S5b).

**Fig. 6:**
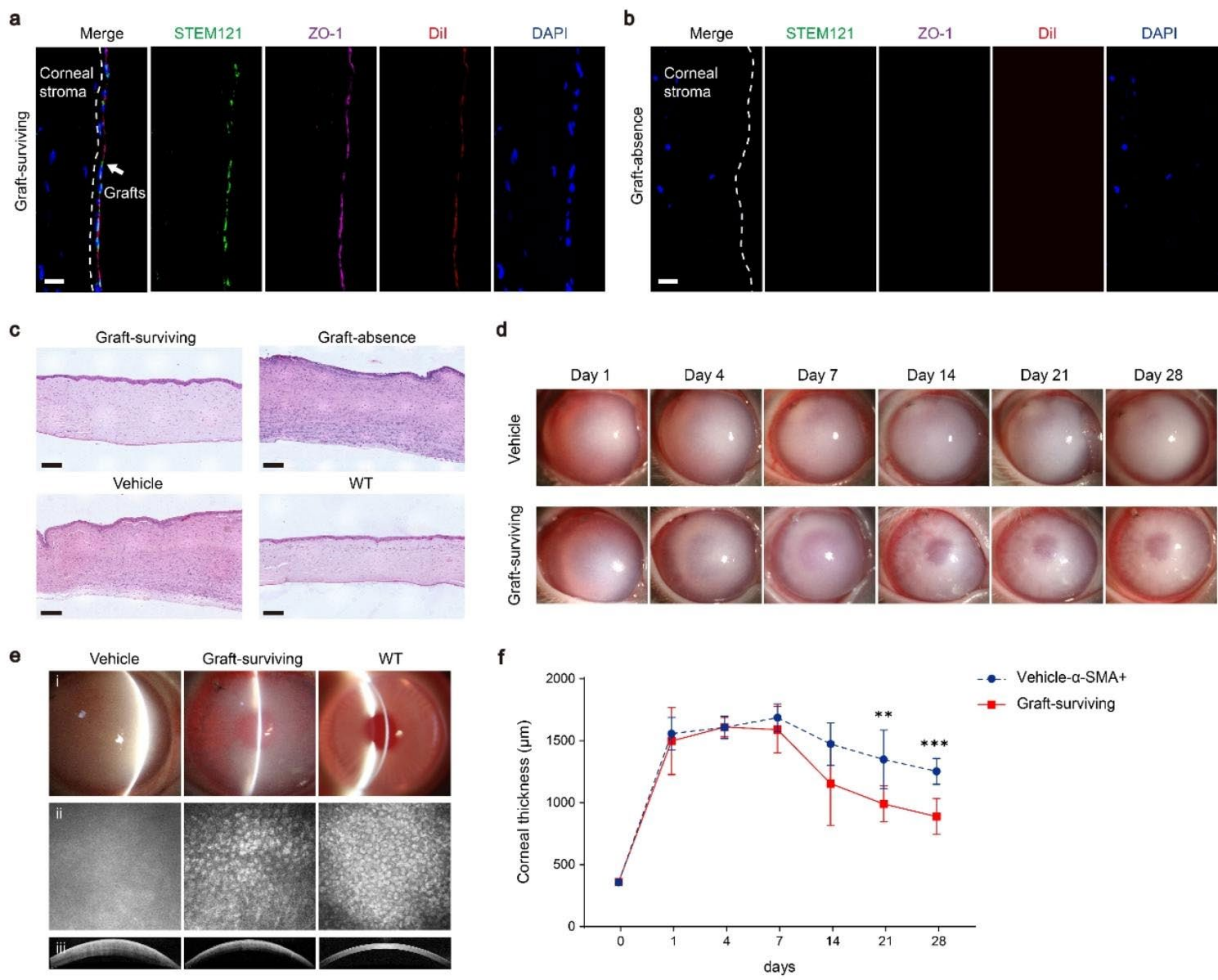
Transplantation of iCEC products improves corneal functional recovery after corneal endothelial dysfunction. **a b** Immunohistochemistry staining showing that the injected iCEC products survived and coexpressed a human-specific marker (STEM 121), corneal endothelial cell marker (ZO-1) and red membrane fluorescence probe (DiI) in the graft-surviving group **a**, while these markers were not expressed in the graft-absence group **b**. Nuclei were stained with DAPI. Scale bar: 50 μm. **c** Representative images of hematoxylin and eosin staining showing that the injected iCEC products were adherent to Descemet’s membrane. The corneal thickness was thinner in the graft-surviving group than in the graft-absence and vehicle groups. Scale bars: 100 μm. **d** The anterior segment images of rabbits showing that corneal transparency was improved with iCEC products injection, while corneal edema was maintained without iCEC products injection from day 1 to day 28 after surgery. **e** Slit-lamp microscopic images (i) showing that corneal clarity was improved after iCEC products transplantation, while corneal opacity was maintained in the vehicle group. confocal scanning laser ophthalmoscopy images (ii) showing that the number and morphology of corneal endothelial cells were recovered in the graft-surviving group, while there were no cells observed in the vehicle group. Visante OCT images (iii) showing that corneal thickness was decreased in the graft-surviving group, while there was no change in the vehicle group. **f** Changes in corneal thickness in the graft-surviving and vehicle groups at different time points after surgery. *n =* 6, ^*^p < 0.05, ^**^p < 0.01, ^***^p < 0.001

Then, iCECs (1 × 10^6^ cells per rabbit) labeled with DiI (a red fluorescence probe) were immediately transplanted into the rabbit anterior chamber (iCECs, n = 12; vehicle control, n = 12). Corneal transparency observation and corneal thickness measurement were evaluated at 1, 4, 7, 14, 21 and 28 days after surgery. No animal died during the postsurgery observation period. By the end of 28 days after surgery, rabbits were euthanized for grafts analysis. In 6 of the 12 rabbits in the graft group, a continuous monolayer of DiI-labeled cells was observed behind Descemet’s membrane in corneal slices. The grafts expressed ZO-1 and the human marker STEM121 (Fig. 6a and Fig. S6), indicating that grafted cells retained the CECs phenotypes in vivo. Moreover, no STEM121-/ZO-1 + cells were observed (Fig. S6b-d). Therefore, we speculated that the transplanted iCECs formed a new corneal endothelium layer in different areas of the cornea. However, there were no human cells or iCECs in the other 6 animals in the graft group (Fig. 6b). In line with this, HE staining also showed a continuous layer of cells attached to the inner side of the cornea in the graft-surviving (n = 6) and WT groups (n = 3) but not in the graft-absence (n = 6) or vehicle groups (Fig. 6c).

To determine the reason for the variable outcomes in cell surviving, we further examined the pathology of the sections. We found that a large number of α-SMA + cells with fibrous morphology were located in the corneas of all the rabbits without grafts (n = 6) (Fig. S7a). In contrast, no SMA + cells were observed in graft-surviving animals (n = 6) (Fig. S7b). Also, we examined α-SMA expression in the vehicle and WT groups. The results showed that the graft group had a similar pattern to the vehicle group, that is, half of the animals underwent stromal fibrosis, while no α-SMA + cells were observed in WT corneas (Fig. S7c-e). Numerous studies have demonstrated that corneal injury, especially the Descemet’s membrane injury may lead to corneal fibrosis and scar formation, which is characterized by the expression of α-SMA [35–39]. HE staining showed that there was a continuous Descemet’s membrane in graft-surviving group, while it was absent in graft-absence group (Fig. 6c). We also found that the vehicle corneas expressing α-SMA lacked Descemet’s membrane and formed fibrosis after injury, but the corneas of the vehicle-α-SMA- group and WT group had continuous Descemet’s membrane (Fig. S7f). These results indicated that the fibrosis of the corneal stroma may be caused by the loss of Descemet’s membrane.

Next, we assessed the effects of iCEC grafts by slit lamp, HRT3 and Visante optical coherence tomography (OCT) examination. We found that the clarity of the cornea decreased dramatically 1 day after surgery. Until day 28, graft-surviving eyes showed a time-dependent recovery of corneal transparency, while the graft-absence and vehicle groups showed no recovery (Fig. 6d). Slit lamp examination revealed that the cornea maintained edema in the vehicle group, while the light transmittance was improved in the graft-surviving group at day 28. Confocal microscopy examination showed polygonal iCECs on Descemet’s membrane in the graft-surviving group, while there were no detectable iCECs in the vehicle group. OCT analysis also showed a decreased corneal thickness in the grafted group relative to the vehicle group (Fig. 6E). We compared differences of corneal thickness between the graft-surviving group (n = 6) and the vehicle-α-SMA + group (n = 6). All rabbits in the graft-surviving group showed gradual improvement after day 7 and marked reduction in corneal thickness at days 21 (^**^p < 0.01) and 28 (^***^p < 0.001). The mean corneal thickness of the graft-surviving group at day 28 was 887.7 ± 144.91 mm, which improved by approximately 32.02% compared with the vehicle (α-SMA+) group (Fig. 6f). These results indicated that corneal edema could be rescued by grafted iCECs.

## Discussion

Human PSC-based replacement therapy is a promising strategy for treating corneal endothelial dysfunction [40]. For the application of cell therapy, it is critical to develop robust differentiation protocols and quality control schemes through the manufacturing process, resulting in quality-controlled therapeutic cell products. In this study, we developed a novel xeno-free protocol for the generation of transplantable human iCECs. We confirmed that SB431542, DKK2 and SU-5402 can efficiently convert NCCs into iCECs within 12 days, leading to improved expression of CECs markers, such as ZO-1, Na^+^ K^+^ ATPase, N-cadherin and CD166 (> 95%). The robustness of this method was further validated by successful differentiation of iCECs with 2 other hESC lines. Such multiple markers would be useful to completely define cell purity in the cell products.

The most important concern of hPSC-based therapy is undifferentiated PSCs in the final products, which may lead to tumorigenicity. We analyzed residual hESCs by immunofluorescence staining and qPCR but detected no pluripotent or proliferative cells. Further in vivo safety analysis was performed with NOG SCID mice, and the results of histological analysis after 6 months showed no teratoma in the injection sites. As additional evidence of the safety of our cells, we did not observe cell proliferation in our rabbit graft models. Another important quality issue facing cell-based therapy is the cell formulation. We also established a robust cryopreservation and cell stability scheme by screening cryopreservation solutions. The development of off-the-shelf iCEC products allows for the storage of a large number of cells in the same batch and extensive testing prior to application. Notably, the serum-free cell cryopreservation medium CELLBANKER 2 had the highest recovery rate and batch-to-batch stability.

The ion pump function of CECs is crucial for driving water movement across the endothelium, which ensures a relatively dehydrated state of the cornea, thereby maintaining clarification [41]. We also established in vitro functional assessment methods to control for batch variation and predict the therapeutic efficacy of iCECs. The results demonstrated that the differentiated iCECs on day 24 were mature iCECs. Our data presented here support prior reports that the membrane potential of cells can be measured by the combination of oscilloscope and Transwells to evaluate cell maturity [31]. Moreover, RNA-seq data identified potential molecular markers of mature iCECs, which may serve as surrogates for predicting their functional properties. Notably, the trend of the potential difference changed significantly after adding ouabain, which further proved the reliability of ensuring cell functional maturation by this method.

We further demonstrated that transplanted iCECs formed a cell layer attached to host tissue, and the corneal transparency was increased, indicating that the iCECs layer functions in the cornea. The results also showed that the graft did not survive in some rabbits, and further analysis revealed the expression of the fibrosis marker α-SMA in the corneas of these animals, suggesting that fibrosis might affect the survival of the grafts. Similar results were seen in other previous reports that found α-SMA expression in corneal endothelial precursor grafts, and the survival and function of grafted cells were largely reduced [26]. Although numerous studies have shown the therapeutic effect of hPSC-derived CECs in rabbit or nonhuman primate models [24–26, 42], the method of modeling in most of them is to scrape off parts of the iCECs of the hosts [25, 26, 42]. In clinical trials of pCECs transplantation, the human corneal endothelium is completely scraped off [8]. In the current study, to enable consistency with clinical applications, we scraped all the rabbit corneal endothelium, which may have caused additional damage. In our vehicle group, α-SMA expression was also found in half of the rabbits, indicating that the fibrosis was caused by scraping of Descemet’s membrane during surgery. Therefore, to develop anti-fibrosis strategies, we established an in vitro cell model as described previously [43]. The results showed that iCECs exhibited fibroblast morphology after treatment with TGFβ1, while SB431542 could effectively inhibit cell fibrosis (Fig. S8). Next, we will further explore the feasibility of SB431542 combination with iCECs transplantation to reduce the effect of corneal stromal fibrosis in the future animal experiments. Moreover, the New Zealand white rabbit has been widely used for corneal endothelial cell degeneration models because it shares similar CECs density, corneal diameter and central corneal thickness with humans. However, rabbit CECs can regenerate after injury, making it difficult to evaluate the efficacy of the tested treatment. In this case, cells need to be transplanted immediately after modeling, which causes the cells to face an acute inflammatory environment. It seems that surgical injury and inflammatory reactions are the main obstacles to the success of iCECs replacement. Therefore, an important issue to be improved in the future is the development of strategies to solve the limited survival of transplant cells, suggesting that further studies are needed to address fibrosis after injury and inflammation.

Overall, our study provides a novel and comprehensive strategy suitable for translational applications of iCECs. Our data also indicate that fibrosis due to surgery might be informative of the treatment outcomes.

## Materials and methods

### Neural crest cell differentiation of hESCs

The hESC lines CB0017, CB0018 and CB0019 were obtained from the National Stem Cell Resource Center, Institute of Zoology, Chinese Academy of Sciences (Beijing, China) and prepared as described previously [44]. The hESCs were maintained on vitronectin-coated (Gibco) plates with E8 medium (Gibco). Cells were passaged every 4–5 days with TrypLE™ Select CTSTM™ (Gibco) to new vitronectin-coated plates. All cells were cultured in a 37 °C and 5% CO_2_ incubator. The culture medium was changed every day.

The NCCs differentiation protocols used in this study are from previous work [45]. Briefly, hESCs in E8 medium were dissociated into single cells with TrypLE™ Select CTS™ and seeded onto vitronectin-coated plates containing E8 medium and 10 µM Y27632 (Selleck) at a density of 6 × 10^4^ cells/cm^2^.

### Differentiation potential of hESC-derived NCCs

For peripheral neurons, hESC-derived NCCs were dissociated into single cells with TrypLE™ Select CTS™ and seeded onto vitronectin-coated plates at a density of 3 × 10^4^ cells/cm^2^ in N2B27 medium. The N2B27 medium consisted of Neurobasal™ Medium CTS™ (Gibco), 1× N-2 Supplement CTS™ (Gibco), 1× CTS™ B-27™ Supplement XenoFree (50×) (Gibco), 10 ng/mL EGF (R&D Systems) and 10 ng/mL bFGF (Peprotech). The next day, the N2B27 medium was switched into peripheral neuron differentiation medium, which contained 1× N-2 Supplement CTS™, 1× CTS™ B-27™ Supplement XenoFree (50×), 200 µM ascorbic acid (AA) and 2 µM dibutyryl-cAMP (dbcAMP) (both from Sigma‒Aldrich), as well as 25 ng/mL brain-derived neurotrophic factor (BDNF), 25 ng/mL neurotrophin-3 (NT3) and 50 ng/mL glial cell line-derived neurotrophic factor (GDNF) (all from Peprotech). Half of the medium was changed every 2–3 days. The expression of peripheral neuron markers was detected at day 20.

For Schwann cell differentiation, hESC-derived NCCs were seeded onto vitronectin-coated plates at a density of 5 × 10^4^ cells/cm^2^ in N2B27 medium for 2 weeks. Then, the medium was changed to N2B27 medium without EGF and bFGF and supplemented with 10 ng/mL ciliary neurotrophic factor (Peprotech), 20 ng/mL neuregulin (R&D Systems) and 0.5 mM dbcAMP. The medium of the cells was changed every 2–3 days. Then, the expression of Schwann cell markers was identified at day 28.

For MSCs differentiation, hESC-derived NCCs were seeded onto vitronectin-coated plates at a density of 1 × 10^4^ cells/cm^2^ in differentiation medium consisting of α-MEM and 10% FBS (both from Gibco) for approximately 3 weeks. For MSCs derivative differentiation, a Human Mesenchymal Stem Cell Functional Identification Kit (R&D Systems) was used. MSC-derived adipogenic cells were stained with Oil Red O (Solarbio) for lipid droplet analysis. MSC-derived osteogenic cells were stained with Alizarin Red (Solarbio) for analysis of mineralized nodule formation. MSC-derived chondrogenic pellets were first embedded in paraffin, cut into 10 μm sections, and then stained with Alcian Blue (Solarbio) to evaluate glycosaminoglycan content.

### Generation of iCECs from hESC-derived NCCs

hESC-derived NCCs were dissociated into single cells with TrypLE™ Select CTS™ and seeded onto vitronectin-coated plates containing iCECs differentiation medium and 10 µM Y27632 at a density of 5 × 10^4^ cells/cm^2^. Basal medium consisted of KNOCKOUT™ DMEM/F-12 CTS™, KNOCKOUT™ SR XenoFree CTS™, 1% GlutaMAX™ CTS™, 1% MEM Non-Essential Amino Acids (NEAA) and 1% Insulin-Transferrin-Selenium (ITS) (all from Gibco), as well as 0.1% ascorbic acid (AA) (Sigma‒Aldrich). iCECs differentiation medium included basal medium and 10 µM SB431542, 10 ng/mL DKK2 (R&D Systems) and 100 nM SU-5402 (Biovision). Cells were cultured in differentiation medium for 12 days. The medium was changed every day.

To induce normal iCECs to fibroblast morphology, we used basal medium supplemented with 10 ng/ml TGFβ1 (Peprotech) to culture normal iCECs for 5 days. Next, replacing TGFβ1 with SB431542 and cultured the iCECs for another 7 days, then evaluated the expression of cell markers.

### Quantitative real-time PCR

RNA extraction was performed using TRIzol (Ambion) according to the manufacturer’s instructions. A NanoDrop ND-1000 spectrophotometer (NanoDrop Technologies) was used to measure the concentration of extracted RNA. RNA was converted into cDNA by a two-step protocol using the PrimeScript 1st strand cDNA synthesis kit (TaKaRa). Real-time PCR was performed using SYBR Green reagent (TOYOBO) on a LightCycler 480 Detection System (Roche Diagnostics). The PCR conditions were 1 min at 95 °C followed by 40 cycles of 95 °C for 15 s and 60 °C for 30 s. The expression of specific genes was normalized to that of *GAPDH*, and the changes in expression were calculated as fold changes by ΔΔCt. All primer sequences are listed in Table S2.

### Flow cytometry

Cells were dissociated into single cells with TrypLE™ Select CTS™ and then resuspended in DPBS (Gibco). For surface marker staining, cells were incubated with 2% BSA (Sigma‒Aldrich) for 30 min. Then, cells were incubated with primary antibody diluted in 100 µL 2% BSA per sample for 30 min as indicated in Table S3. After washing with DPBS 3 times, the cells were incubated in 2% BSA-diluted secondary antibody for 45 min. The cells were washed with DBPS 3 times, and then the samples were run on a cytometer for analysis. For intracellular marker staining, cells were first fixed in 4% paraformaldehyde (PFA) for 10 min. Primary and secondary antibodies were diluted in 2% BSA and 0.3% Triton (Invitrogen) instead. Appropriate isotype-matched monoclonal antibody controls were selected to identify subset gating and positive populations.

### Immunofluorescence staining

Cells were fixed in 4% PFA at room temperature for 30 min and washed 3 times with DPBS. Cells were treated with 2% BSA (surface primary/secondary antibody diluent) or 2% BSA and 0.3% Triton (intracellular primary/secondary antibody diluent) at room temperature for 2 h. Then, cells were incubated with diluted primary antibodies overnight at 4 °C. On the next day, the cells were washed 3 times with DPBS and incubated with diluted secondary antibodies for 2 h at room temperature. Then, the cells were washed 3 times with DPBS and stained with DAPI (Invitrogen) for 10 min. Samples were affixed to glass slides dripped with anti-fluorescence attenuation solution and fixed with clear nail polish.

The corneal tissue was cut into several parts and embedded with optimal cutting temperature compound after being treated with 4% PFA. The samples were sliced into 10 μm-thick sections and then used for immunofluorescence staining. The expression of markers was observed by confocal laser-scanning microscopy (LSM-880, Carl Zeiss). All antibodies used are listed in Table S4.

### RNA sequencing and analysis

Total RNA from samples (different time points of CEC differentiated cells) harvested from monolayers or microcarriers was extracted using TRIzol. RNA-seq libraries were built for Illumina® using the NEBNext®Ultra™ RNA Library Prep Kit. Sequencing was performed on an Illumina HiSeq X-Ten sequencer with a 150 bp paired-end sequencing reaction. After the sequencing data were filtered, they were mapped to the reference genome hg38 using STAR. Gene expression levels were estimated by counting sequencing sequences (reads) mapped to genomic regions or exon regions and FPKM (Fragments per Kilobase of exon model per Million Mapped Fragments). DESeq2 was used for DEGs analysis, and the screening criteria for DEGs in this project were |log2-fold change| ≥ 1 and *p* value < 0.05. PCA of RNA-seq data from differentiated iCECs at different time points was performed using the prcomp function in R. The heatmap and volcano map were generated with the pheatmap and ggscatter functions in R. The Pearson correlation coefficient was calculated by cor.test in R. Gene Ontology pathway analysis for DEGs was performed with the clusterProfiler package. The pCECs data was obtained from the GEO database (GSE41616). RNA sequencing data of iCECs, hESCs and NCCs were analyzed to identify DEGs using a fold change threshold of 50. DEGs were classified as upregulated or downregulated and GSEA was performed to determine the enrichment of these gene sets in pCECs. Significant enrichment was defined as |normalized enrichment score (NES)| > 1, *p* value < 0.05 and false discovery rate (FDR) < 0.25.

### Hematoxylin & eosin staining

The corneal tissues were embedded in paraffin and sliced into 10 μm-thick sections. After paraffin removal with xylene (Aladdin), the sample was hydrated with gradient concentrations of ethanol. The samples were washed with tap water and stained with hematoxylin (ZSGB-BIO), washed with 0.1% aqueous ammonia, and finally stained with eosin (ZSGB-BIO). The samples were dehydrated by gradient ethanol, treated with xylene and affixed to cover slips dripped with neutral balsam. The tissues were observed with a Leica Aperio VERSA8 Fluorescent Slide Scanner.

### Transendothelial electrical potential difference recordings

hESC-derived NCCs were seeded onto a vitronectin-coated Transwell (Corning) insert in plates containing iCECs differentiation medium. Cells were washed with DBPS 3 times before recording. The black (or reference) alligator clip was connected to a copper electrode, which was inserted into the lower compartment. The red (or positive) alligator clip was connected to a cooper electrode, which was inserted into the upper compartment. Alligator clips connected to the wires, respectively connected to an oscilloscope (Tektronix), red channel 1, black channel 2. At this time, iCECs differentiation medium was added to the upper and lower compartments. The oscilloscope was turned on and set to 10 times per second for a total of 2 h. Control hESCs were also seeded onto Transwell inserts in a plate for 5 days, and the potential difference was measured. The final potential difference was calculated by subtracting black channel 2 from red channel 1.

The potential difference of iCECs on the day 24 of differentiation was recorded. After the potential difference was stable, 10 µM of the Na^+^ K^+^ ATPase inhibitor ouabain (Sigma‒Aldrich) was added to the upper compartment, and then the change in potential difference was observed.

### iCECs cryopreservation and thawing

For iCECs cryopreservation, cells were dissociated into single cells with TrypLE™ Select CTS™ and counted with CountStar Rigel S5. Then, the cells were resuspended in freezing solutions and divided into cryogenic vials (Corning). All cryogenic vials were placed in CoolCell® containers (Corning), placed in a -80℃ refrigerator (Thermo Fisher) for gradient cooling, and transferred to liquid nitrogen the next day. For iCECs thawing, the cells were thawed with an automated thawing system (BioLife) and immediately added to warm medium. After centrifugation, the supernatant was discarded, and the thawed iCECs were washed 3 times with DPBS. To determine the viability of iCECs after thawing, we resuspended cells in injection solution, placed them at 4 °C for 6 h, and then measured the cell viability with a cell counter every 2 h to simulate the cell transplantation procedure.

### Teratoma formation

For the in vivo safety study, 7-week-old male NOG SCID mice from Vital River Laboratory Animal Technology Co., Ltd. were used. iCECs were dissociated into single cells, and then 100 µL solutions containing 1 × 10^6^ cells and ice-cold Matrigel (Corning) were injected into the subcutaneous space of the back of NOG SCID mice (n = 20). Additionally, 1 × 10^6^ control cells (hESC line CB0019) were injected into NOG SCID mice (n = 10). All mice injected with iCECs were observed for 6 months. The subcutaneous tumors were fixed with 4% PFA, paraffin embedded and sliced at 5 μm for staining.

### Cell transplantation

DiI staining solution (1 mM DiI:differentiation medium = 1:200) was added to the iCECs 3 days before transplantation and incubated at 37 °C for 6 h. The cells were washed with warm medium 3 times and observed with a confocal laser-scanning microscope to determine whether the staining was successful. The rabbit in vivo study following GLP regulations was performed at JOINN Laboratories (Suzhou) Inc., Suzhou, China. The animals were kept at 18–26 °C, relative humidity 40–70%, good ventilation and alternating light and dark for 12 h. Male New Zealand white rabbits 3–4 months old weighing 2.0-2.5 kg were used. Tacrolimus eye drops (Senju Pharmaceutical Co., Ltd) were given to animals 3 days before the operation, 3 times a day. On the day of operation, the animals were anesthetized intramuscularly with ketamine hydrochloride (50 mg/mL, 10 mg/kg) and xylazine hydrochloride (20 mg/mL, 3.2 mg/kg). Topical anesthesia was performed with benoxinate hydrochloride during the operation. All rabbits were randomly divided into two groups (n = 12/group). The right eye was the operative eye. After eye disinfection, the anterior chamber was incised with a 1.2 mm slit knife at 9 o’clock, and the corneal endothelium was completely scraped off from Descemet’s membrane with a homemade 20G silicone needle. The residual corneal endothelial cells in the anterior chamber were rinsed slowly with sodium lactate Ringer’s injection. During debridement, air was injected to maintain the anterior chamber. The lenses of all rabbits were preserved.

The iCECs were dissociated into single cells. Then, 1 × 10^6^ cells were suspended in 100 µL iCECs basal medium supplemented with 10 µM Y27632. The cell suspension was injected into the center of the anterior chamber with a clean 30G insulin needle. The rabbit was turned and the eye was kept down for at least 3 h to ensure cell attachment. Tacrolimus eye drops were given daily for 1 week, and levofloxacin eye drops and tobramycin dexamethasone ophthalmic ointment were given daily for 2 weeks after the operation. The vehicle eyes were injected with iCECs basal medium supplemented with 10 µM Y27632.

The anterior segment of the eyes and corneal transparency were examined with a slit lamp, the central corneal thickness was measured by OCT, and the central corneal endothelial cells were observed by confocal scanning laser ophthalmoscopy on days 1, 3, 5, 7, 14, 21, and 28 after surgery. Photos were collected and saved at each inspection point. The measured data had three readings at a time, and the average value was taken.

### In vivo tracing of iCECs

Luciferase-labeled hESC line (CB0019) was prepared as described previously [46]. The differentiation protocol of luciferase-labeled iCECs has been mentioned above. Male New Zealand white rabbits 3–4 months old weighing 2.0-2.5 kg were used. 1 × 10^6^ luciferase-labeled iCECs were suspended in 100 µL iCECs basal medium supplemented with 10 µM Y27632. And the cell suspension was injected into the center of the anterior chamber. Then, the rabbit was anesthetized intramuscularly with ketamine hydrochloride and xylazine hydrochloride at day 14, 21, 28 after surgery. Immediately after anterior chamber administration of 100 µL of 30 µM Akalumine-HCl (TokeOni), bioluminescent images of eyes were acquired using IVIS Lumina III (PerkinElmer). The images were analyzed by Living Image 4.3 software (PerkinElmer).

### Statistical analysis

All experiments were performed using at least 3 biological replicates. The data are presented as the mean ± SD. Statistical analysis was performed by using GraphPad Prism 8 and OriginPro 2022 software. ImageJ 1.52a and ImageScope x64 were used to analyze immunofluorescence staining and HE staining, respectively. Flow cytometry results analysis was performed by using CytExpert 2.3 and FlowJo V10.0. The statistical significance (*p* value) of differences between mean values was determined using Student’s *t* test, one-way analysis of variance (ANOVA) or two-way ANOVA. ^*^p < 0.05 was considered significant.

### Electronic supplementary material

Below is the link to the electronic supplementary material.

Supplementary Material 1. **Additional file 1. Figure ****S1****.** Generation of NCCs from multiple hESC lines. **Figure S2.** Differentiation of multiple hESC-derived NCCs into multiple iCECs. **Figure S3.** Global expression profiles of the cells during iCECs differentiation of hESCs. **Figure S4.** In vivo tracing of iCECs. **Figure S5.** Establishment of animal model of corneal endothelial dysfunction. **Figure S6.** Transplanted iCECs was observed in different areas of cornea. **Figure S7.** Analysis of iCEC products survival in the graft group 28 days after surgery. **Figure S8.** SB431542 can rescue iCECs fibrosis. **Table ****S1****.** Quality control and in-process tests for the manufacturing of iCECs. **Table S2.** Primers used for qPCR. **Table S3.** List of antibodies used in FACS. **Table S4.** Antibodies used in Immunofluorescence Staining

## Data Availability

All data and materials in this study are available upon reasonable request.

## List of abbreviations

hPSCs: human pluripotent stem cells
hESCs: human embryonic stem cells
NCCs: neural crest cells
CECs: corneal endothelial cells
iCECs: induced corneal endothelial cells
pCECs: primary corneal endothelial cells
MSCs: mesenchymal stem cells
FBS: fetal bovine serum
dbcAMP: dibutyryl-cAMP
BDNF: brain-derived neurotrophic factor
NT3: neurotrophin-3
GDNF: glial cell line-derived neurotrophic factor
NEAA: Non-Essential Amino Acids
ITS: Insulin-Transferrin-Selenium
AA: ascorbic acid
PFA: paraformaldehyde
GO: Gene Ontology
FPKM: Fragments per Kilobase of exon model per Million Mapped Fragments
GSEA: gene set enrichment analysis
ES: enrichment score
NES: normalized enrichment score
FDR: false discovery rate
OCT: optical coherence tomography
GLP: good laboratory manufacturing practice
QC: quality control

## References

[1] Maurice DM. The structure and transparency of the cornea. J Physiol. 1957;136(2). 10.1113/jphysiol.1957.sp005758. 263 – 86. 10.1113/jphysiol.1957.sp005758 PMC1358888 13429485

[2] Maurice DM (1972). The location of the fluid pump in the cornea. J Physiol.

[3] Bonanno JA (2012). Molecular mechanisms underlying the corneal endothelial pump. Exp Eye Res.

[4] Tan DT, Dart JK, Holland EJ, ,Kinoshita S (2012). Corneal transplantation. Lancet.

[5] Bourne WM, Nelson LR, ,Hodge DO (1997). Central corneal endothelial cell changes over a ten-year period. Invest Ophthalmol Vis Sci.

[6] Sherrard ES. The corneal endothelium in vivo: its response to mild trauma. Exp Eye Res. 1976;22(4). 10.1016/0014-4835(76)90227-x. 347 – 57. 10.1016/0014-4835(76)90227-x 954871

[7] Gain P, Jullienne R, He Z, Aldossary M, Acquart S, Cognasse F (2016). Global survey of corneal transplantation and Eye Banking. JAMA Ophthalmol.

[8] Kinoshita S, Koizumi N, Ueno M, Okumura N, Imai K, Tanaka H (2018). Injection of cultured cells with a ROCK inhibitor for Bullous Keratopathy. N Engl J Med.

[9] Numa K, Imai K, Ueno M, Kitazawa K, Tanaka H, Bush JD (2021). Five-year follow-up of First 11 patients undergoing injection of cultured corneal endothelial cells for corneal endothelial failure. Ophthalmology.

[10] Thomson JA, Itskovitz-Eldor J, Shapiro SS, Waknitz MA, Swiergiel JJ, Marshall VS (1998). Embryonic stem cell lines derived from human blastocysts. Science.

[11] Takahashi K, Tanabe K, Ohnuki M, Narita M, Ichisaka T, Tomoda K (2007). Induction of pluripotent stem cells from adult human fibroblasts by defined factors. Cell.

[12] Yu J, Vodyanik MA, Smuga-Otto K, Antosiewicz-Bourget J, Frane JL, Tian S (2007). Induced pluripotent stem cell lines derived from human somatic cells. Science.

[13] Zhang K, Pang K, ,Wu X (2014). Isolation and transplantation of corneal endothelial cell-like cells derived from in-vitro-differentiated human embryonic stem cells. Stem Cells Dev.

[14] McCabe KL, Kunzevitzky NJ, Chiswell BP, Xia X, Goldberg JL, ,Lanza R (2015). Efficient generation of human embryonic stem cell-derived corneal endothelial cells by Directed differentiation. PLoS ONE.

[15] Song Q, Yuan S, An Q, Chen Y, Mao FF, Liu Y (2016). Directed differentiation of human embryonic stem cells to corneal endothelial cell-like cells: a transcriptomic analysis. Exp Eye Res.

[16] Zhao JJ, Afshari NA (2016). Generation of human corneal endothelial cells via in Vitro Ocular lineage restriction of pluripotent stem cells. Invest Ophthalmol Vis Sci.

[17] Zhang C, Du L, Sun P, Shen L, Zhu J, Pang K (2017). Construction of tissue-engineered full-thickness cornea substitute using limbal epithelial cell-like and corneal endothelial cell-like cells derived from human embryonic stem cells. Biomaterials.

[18] Chen X, Wu L, Li Z, Dong Y, Pei X, Huang Y (2018). Directed differentiation of human corneal endothelial cells from human embryonic stem cells by using cell-conditioned culture media. Invest Ophthalmol Vis Sci.

[19] Wagoner MD, Bohrer LR, Aldrich BT, Greiner MA, Mullins RF, Worthington KS, et al. Feeder-free differentiation of cells exhibiting characteristics of corneal endothelium from human induced pluripotent stem cells. Biol Open. 2018;7(5). 10.1242/bio.032102. 10.1242/bio.032102 PMC5992532 29685994

[20] Ali M, Khan SY, Kabir F, Gottsch JD, ,Riazuddin SA (2018). Comparative transcriptome analysis of hESC- and iPSC-derived corneal endothelial cells. Exp Eye Res.

[21] Van Horn DL, Sendele DD, Seideman S, ,Buco PJ (1977). Regenerative capacity of the corneal endothelium in rabbit and cat. Invest Ophthalmol Vis Sci.

[22] Koizumi N, Sakamoto Y, Okumura N, Okahara N, Tsuchiya H, Torii R (2007). Cultivated corneal endothelial cell sheet transplantation in a primate model. Invest Ophthalmol Vis Sci.

[23] Shen L, Sun P, Zhang C, Yang L, Du L, ,Wu X (2017). Therapy of corneal endothelial dysfunction with corneal endothelial cell-like cells derived from skin-derived precursors. Sci Rep.

[24] Hatou S, Sayano T, Higa K, Inagaki E, Okano Y, Sato Y (2021). Transplantation of iPSC-derived corneal endothelial substitutes in a monkey corneal edema model. Stem Cell Res.

[25] Ali M, Khan SY, Gottsch JD, Hutchinson EK, Khan A, ,Riazuddin SA (2021). Pluripotent stem cell-derived corneal endothelial cells as an alternative to donor corneal endothelium in keratoplasty. Stem Cell Reports.

[26] Li Z, Duan H, Jia Y, Zhao C, Li W, Wang X, et al. Long-term corneal recovery by simultaneous delivery of hPSC-derived corneal endothelial precursors and nicotinamide. J Clin Invest. 2022;132(1). 10.1172/jci146658. 10.1172/JCI146658 PMC8718141 34981789

[27] Saika S, Saika S, Liu CY, Azhar M, Sanford LP, Doetschman T (2001). TGFbeta2 in corneal morphogenesis during mouse embryonic development. Dev Biol.

[28] Zacharias AL, Gage PJ (2010). Canonical Wnt/beta-catenin signaling is required for maintenance but not activation of Pitx2 expression in neural crest during eye development. Dev Dyn.

[29] Senoo T, Joyce NC (2000). Cell cycle kinetics in corneal endothelium from old and young donors. Invest Ophthalmol Vis Sci.

[30] Lee JG, Jung E, ,Heur M (2018). Fibroblast growth factor 2 induces proliferation and fibrosis via SNAI1-mediated activation of CDK2 and ZEB1 in corneal endothelium. J Biol Chem.

[31] Olszewski C, Maassen J, Guenther R, Skazik-Voogt C, ,Gutermuth A (2022). Mechanotransductive differentiation of hair follicle stem cells derived from aged eyelid skin into corneal endothelial-like cells. Stem Cell Rev Rep.

[32] Gkountela S, Castro-Giner F, Szczerba BM, Vetter M, Landin J, Scherrer R (2019). Circulating Tumor Cell Clustering shapes DNA methylation to Enable Metastasis Seeding. Cell.

[33] Piao J, Zabierowski S, Dubose BN, Hill EJ, Navare M, Claros N (2021). Preclinical efficacy and safety of a human embryonic stem cell-derived midbrain dopamine progenitor product, MSK-DA01. Cell Stem Cell.

[34] Okumura N, Koizumi N, Ueno M, Sakamoto Y, Takahashi H, Tsuchiya H (2012). ROCK inhibitor converts corneal endothelial cells into a phenotype capable of regenerating in vivo endothelial tissue. Am J Pathol.

[35] Masur SK, Dewal HS, Dinh TT, Erenburg I, ,Petridou S (1996). Myofibroblasts differentiate from fibroblasts when plated at low density. Proc Natl Acad Sci USA.

[36] Jester JV, Barry-Lane PA, Cavanagh HD, ,Petroll WM (1996). Induction of alpha-smooth muscle actin expression and myofibroblast transformation in cultured corneal keratocytes. Cornea.

[37] Marino GK, Santhiago MR, Santhanam A, Lassance L, Thangavadivel S, Medeiros CS (2017). Epithelial basement membrane injury and regeneration modulates corneal fibrosis after pseudomonas corneal ulcers in rabbits. Exp Eye Res.

[38] Lassance L, Marino GK, Medeiros CS, Thangavadivel S, ,Wilson SE (2018). Fibrocyte migration, differentiation and apoptosis during the corneal wound healing response to injury. Exp Eye Res.

[39] Medeiros CS, Saikia P, de Oliveira RC, Lassance L, Santhiago MR, ,Wilson SE (2019). Descemet’s membrane modulation of posterior corneal fibrosis. Invest Ophthalmol Vis Sci.

[40] Price MO, Mehta JS, Jurkunas UV, ,Price FW (2021). Corneal endothelial dysfunction: evolving understanding and treatment options. Prog Retin Eye Res.

[41] DelMonte DW, Kim T. Anatomy and physiology of the cornea. J Cataract Refract Surg. 2011;37(3). 10.1016/j.jcrs.2010.12.037. 588 – 98. 10.1016/j.jcrs.2010.12.037 21333881

[42] Pan SH, Zhao N, Feng X, Jie Y, ,Jin ZB. Conversion of mouse embryonic fibroblasts into neural crest cells and functional corneal endothelia by defined small molecules. Sci Adv. 2021;7(23). 10.1126/sciadv.abg5749. 10.1126/sciadv.abg5749 PMC8177713 34088673

[43] Okumura N, Kay EP, Nakahara M, Hamuro J, Kinoshita S, ,Koizumi N (2013). Inhibition of TGF-β signaling enables human corneal endothelial cell expansion in vitro for use in regenerative medicine. PLoS ONE.

[44] Gu Q, Wang J, Wang L, Liu ZX, Zhu WW, Tan YQ (2017). Accreditation of Biosafe Clinical-Grade Human Embryonic stem cells according to Chinese regulations. Stem Cell Reports.

[45] Barber K, Studer L, ,Fattahi F (2019). Derivation of enteric neuron lineages from human pluripotent stem cells. Nat Protoc.

[46] Iwano S, Sugiyama M, Hama H, Watakabe A, Hasegawa N, Kuchimaru T (2018). Single-cell bioluminescence imaging of deep tissue in freely moving animals. Science.

