# Supplementary material for: Safety and efficacy of human ESC-derived corneal endothelial cells for corneal endothelial dysfunction
Source: Cell Biosci. 2023 Nov 6;13:201. doi: 10.1186/s13578-023-01145-w (PMC10629087; doi:10.1186/s13578-023-01145-w)
Supplement: Supplementary file 1 — Supplementary Material 1. Additional file 1. Figure S1. Generation of NCCs from multiple hESC lines. Figure S2. Differentiation of multiple hESC-derived NCCs into multiple iCECs. Figure S3. Global expression profiles of the cells during iCECs differentiation of hESCs. Figure S4. In vivo tracing of iCECs. Figure S5. Establishment of animal model of corneal endothelial dysfunction. Figure S6. Transplanted iCECs was observed in different areas of cornea. Figure S7. Analysis of iCEC products survival in the graft group 28 days after surgery. Figure S8. SB431542 can rescue iCECs fibrosis. Table S1. Quality control and in-process tests for the manufacturing of iCECs. Table S2. Primers used for qPCR. Table S3. List of antibodies used in FACS. Table S4. Antibodies used in Immunofluorescence Staining [file 13578_2023_1145_MOESM1_ESM.docx]

**Supplementary Information**

**Safety and efficacy of human ESC-derived corneal endothelial cells for corneal endothelial dysfunction**

Juan Yu^1,3,5,6†^, Nianye Yu^1,3,5,6†^, Yao Tian^2,3,5,6†^, Yifan Fang^7†^, Bin An^2,5^, Guihai Feng^2,3,4,5^, Jun Wu^2,3,4,5^, Liu Wang^2,3,4,5^, Jie Hao^2,3,4,5^, Liqiang Wang^7^, Qi Zhou^2,3,4,5^, Wei Li^2,3,4,6*^, Yukai Wang^2,3,4,5*^ and Baoyang Hu^1,2,3,4,6*^

^1^ Savaid Medical School, University of Chinese Academy of Sciences, Beijing 100049, China.

^2^ State Key Laboratory of Stem Cell and Reproductive Biology, Institute of Zoology, Chinese Academy of Sciences, Beijing 100101, China.

^3^ Institute of Stem Cell and Regeneration, Chinese Academy of Sciences, Beijing 100101, China.

^4^ Beijing Institute for Stem Cell and Regenerative Medicine, Beijing, China.

^5^ National Stem Cell Resource Center, Chinese Academy of Sciences, Beijing 100101, China.

^6^ University of Chinese Academy of Sciences, Beijing 100864, China.

^7^ Department of Ophthalmology, The First Center of the PLA General Hospital, Beijing, China.

**Supplemental figures**

**
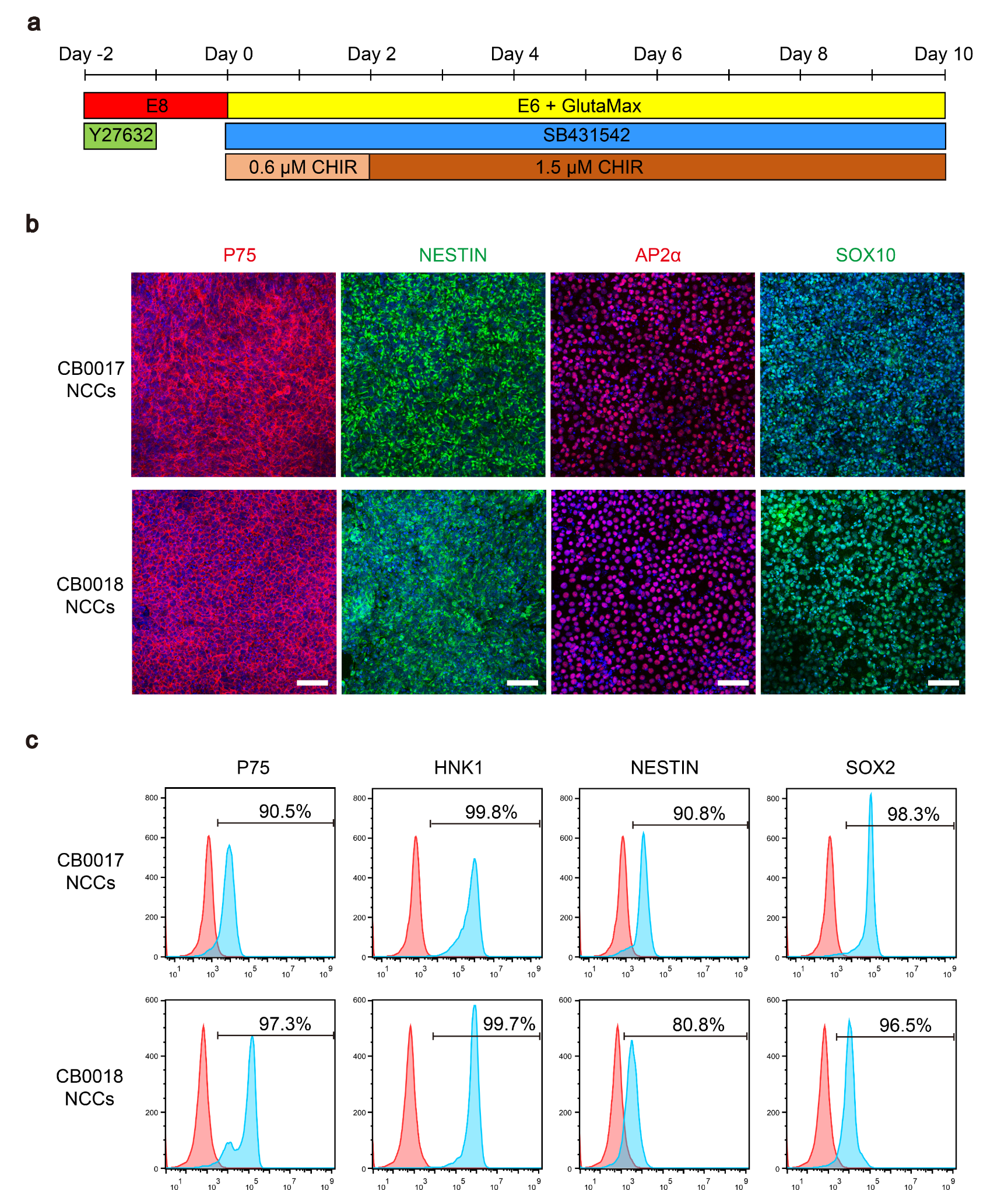
**

**Fig. S1** Generation of NCCs from multiple hESC lines. **a** Schematic illustration of the differentiation conditions used to generate NCCs from multiple hESC lines. **b** Immunofluorescence staining showing that NCCs differentiated from hESC lines (CB0017 and CB0018) expressed P75, NESTIN, AP2α, and SOX10. Nuclei were stained with DAPI. Scale bars: 50 μm. **c** Flow cytometry analysis of P75, HNK1, Nestin and SOX2 expression in NCCs differentiated from hESC lines (CB0017 and CB0018). *n =* 3


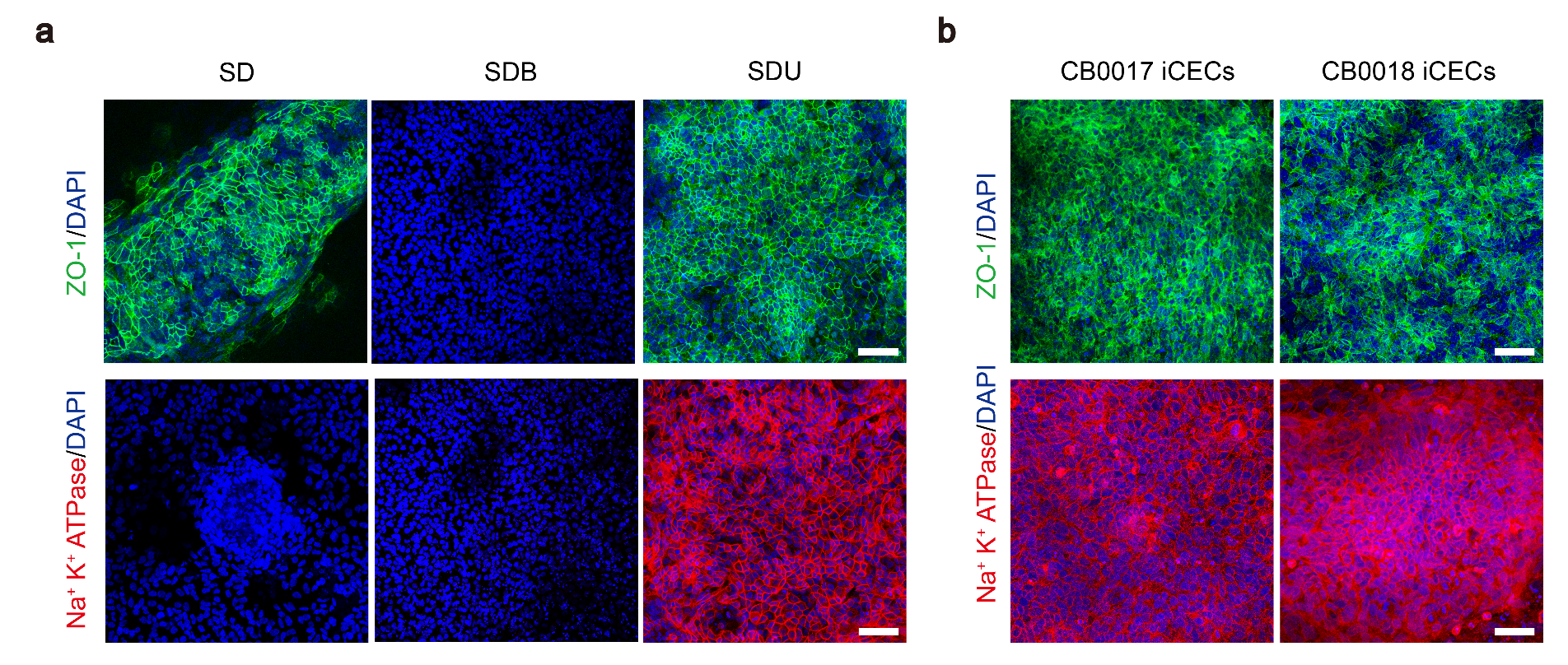


**Fig. S2** Differentiation of multiple hESC-derived NCCs into multiple iCECs. **a** Immunofluorescence staining images of small molecule screen of ZO-1 and Na^+^ K^+^ ATPase expression in iCECs differentiated from CB0019-NCCs. Nuclei were stained with DAPI. Scale bars: 50 μm. **b** Immunofluorescence staining showing that iCECs differentiated from hESC lines (CB0017 and CB0018)-derived NCCs expressed ZO-1 and Na^+^ K^+^ ATPase. Nuclei were stained with DAPI. Scale bars: 50 μm


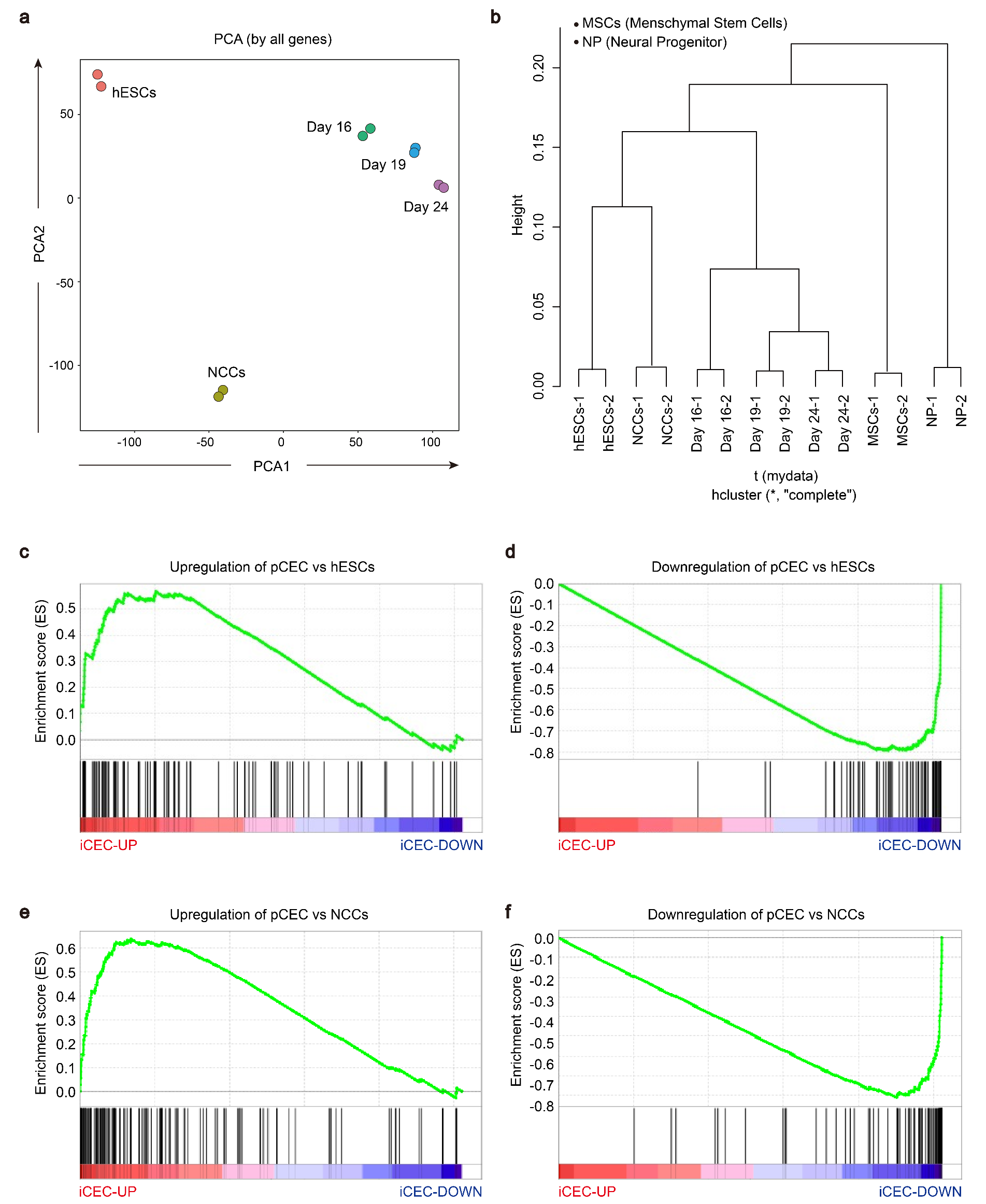


**Fig. S3** Global expression profiles of the cells during iCECs differentiation of hESCs. **a** PCA was carried out to evaluate the similarities of the gene expression profiles between undifferentiated hESCs, NCCs and differentiated iCECs at different time points. **b** Cluster dendrogram analysis for RNA expression among undifferentiated hESCs, NCCs, different time points of iCECs, mesenchymal stem cells (MSCs) and neural progenitor (NP). *n =* 2. **c-f** GSEA enrichment analysis. **c d** Schematic representation of the expression of upregulated gene sets **c** and downregulated gene sets **d** of pCEC relative to hESCs in differentiated iCECs based on RNA-seq studies. **e f** Schematic representation of the expression of upregulated gene sets **e** and downregulated gene sets **f** of pCEC relative to NCCs in differentiated iCECs based on RNA-seq studies


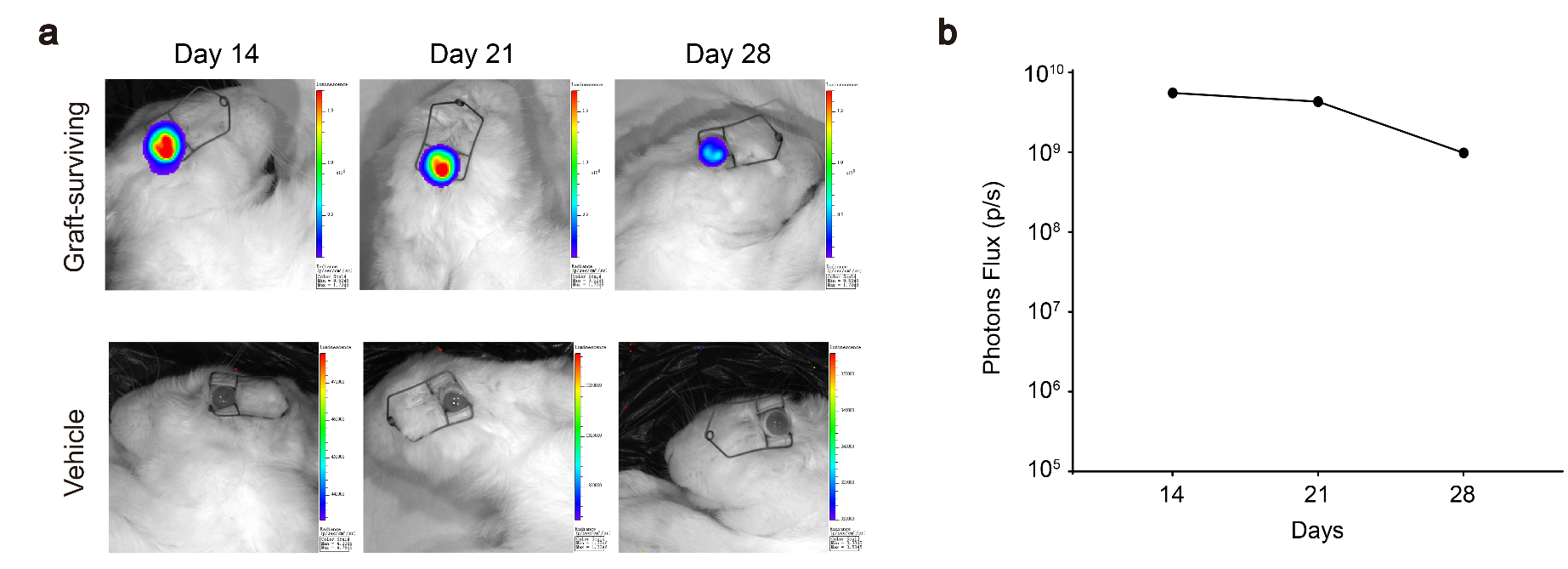


**Fig. S4** *In vivo* tracing of iCECs. **a** Representative images of graft-surviving and vehicle groups with substantial bioluminescent signals. The color bars indicate the total bioluminescence radiance (photons/sec/cm^2^/sr). **b** Quantification of substantial bioluminescent signals in graft-surviving group

**Fig. S5** Establishment of animal models of corneal endothelial dysfunction. **a** Alizarin red staining of rabbit corneas immediately after surgery and WT corneas. Scale bars: 200 μm. **b** HRT3 detection of vehicle groups 1 day postsurgery. Rabbit sample numbers are indicated inside. *n*=6


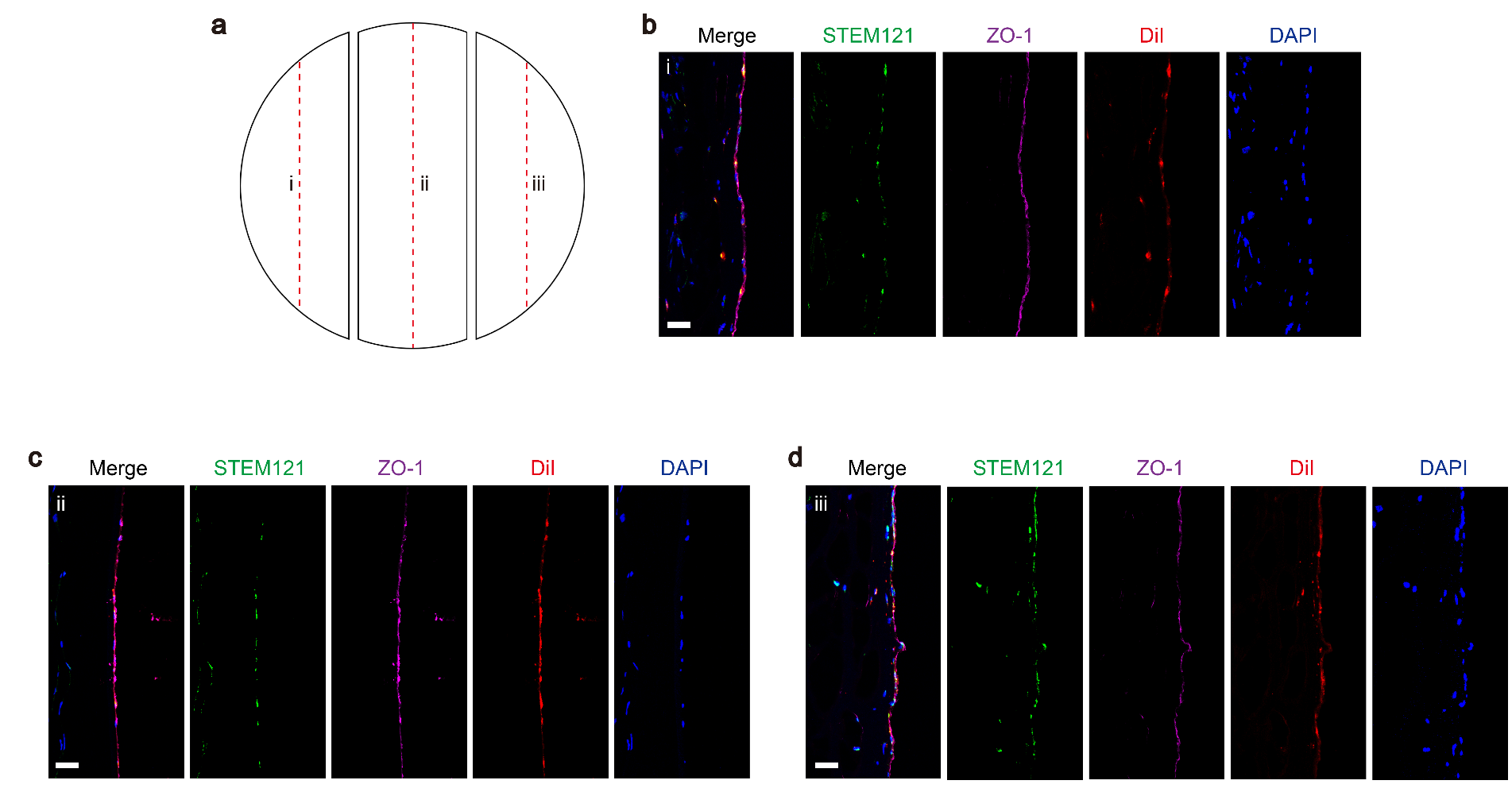


**Fig. S6** Transplanted iCECs was observed in different areas of cornea. **a** Schematic diagram of corneal sample segmentation. The whole cornea is evenly divided into three parts, from left to right being i, ii, iii. The red dotted line represents the staining position of **b-d** in the central cornea of each part. **b-d** Immunofluorescence staining showing that the injected iCECs survived and coexpressed a human-specific marker (STEM 121), corneal endothelial cell marker (ZO-1) and red membrane fluorescence probe (DiI) in central parts of i, ii and iii corneas. Nuclei were stained with DAPI. Scale bar: 50 μm. *n* = 6


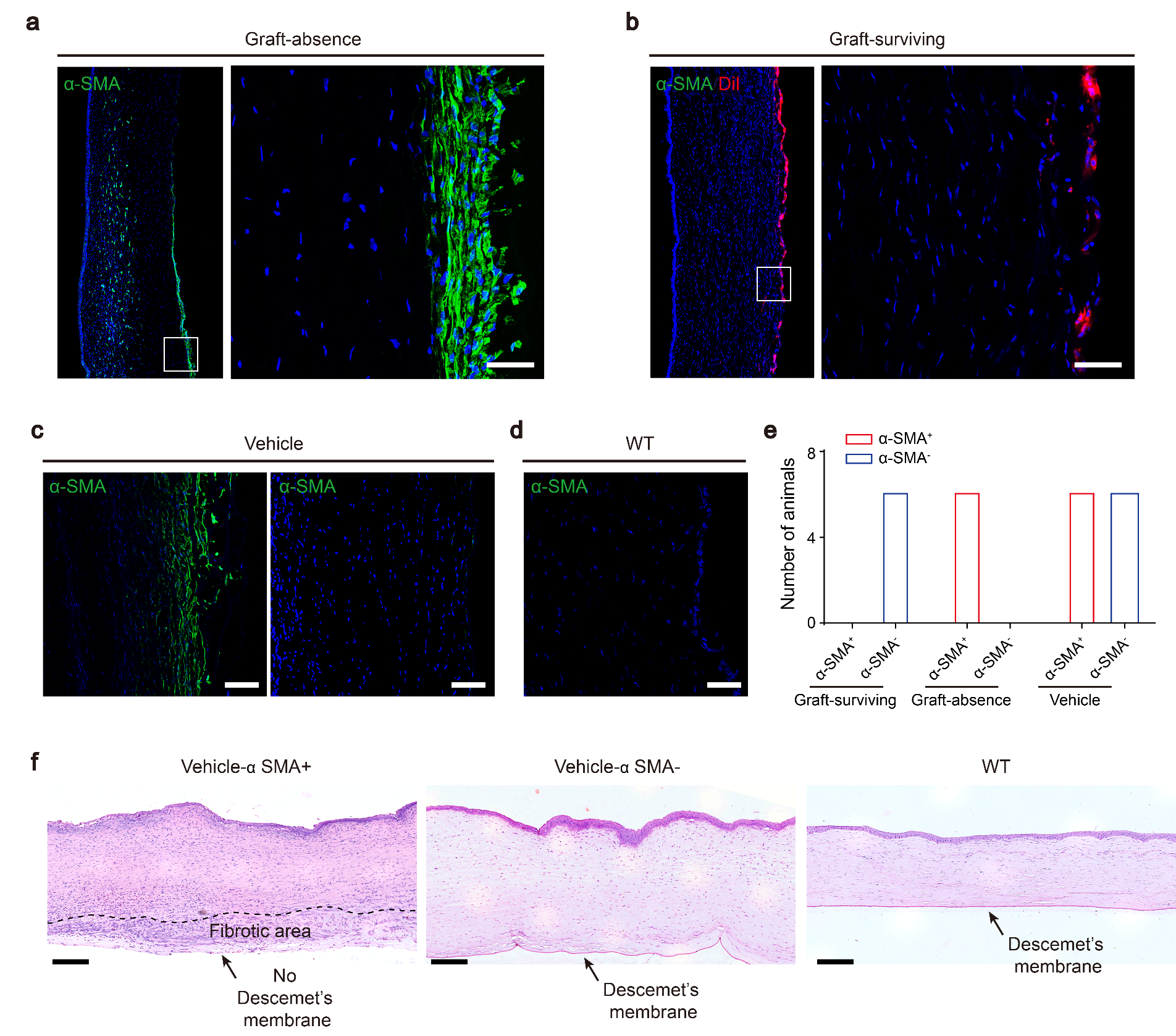


**Fig. S7** Analysis of iCEC products survival in the graft group 28 days after surgery. **a** – **d** The expression of α-SMA in graft, vehicle and WT corneas. **a** Immunofluorescence staining showing that the edge of the corneal stroma in the graft-absence group expressed α-SMA, which indicated no surviving cells, while **b** red DiI was observed in the graft-surviving group, in which iCECs survived. **c** Some corneas expressed α-SMA, while others did not in the vehicle group. **d** No expression of α-SMA in WT corneas. Nuclei were stained with DAPI. Rectangles indicate areas of magnification shown in the right panels. Scale bars: 50 μm. **e** Quantification of α-SMA expression in graft and vehicle groups. **f** Representative images of HE staining showing that there was no Descemet's membrane in vehicle-α SMA+ corneas, while vehicle-α SMA- and WT corneas have continuous Descemet's membrane. Scale bars: 100 μm. *n*=6


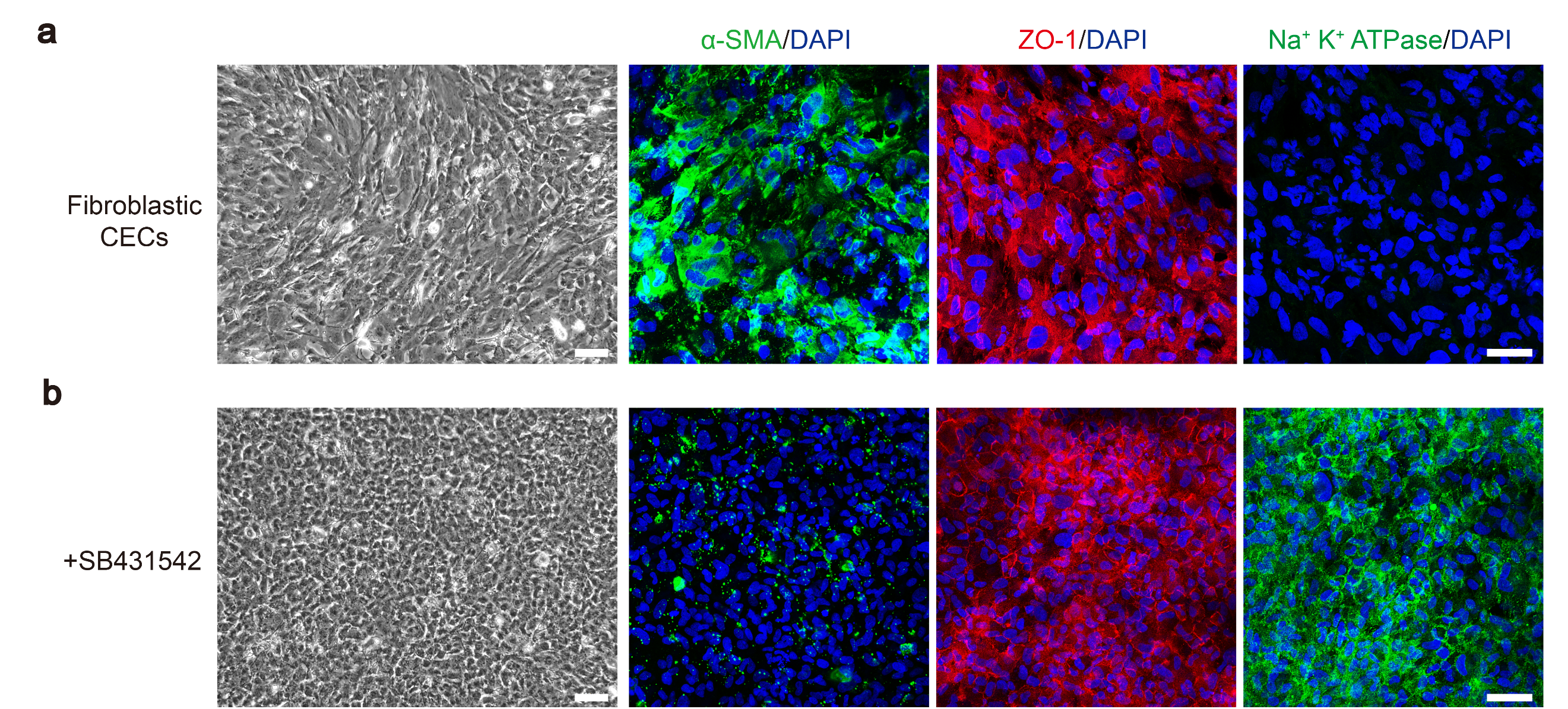


**Fig. S8** SB431542 can rescue iCECs fibrosis. **a** iCECs cultured with 10 ng/ml TGFβ1 exhibited the fibroblastic morphology. Scale bar: 100 μm. And the immunofluorescence staining showing that the fibroblastic iCECs highly expressed fibrosis marker (α-SMA), while didn’t express CEC marker (Na^+^ K^+^ ATPase). Nuclei were stained with DAPI. Scale bar: 50 μm. **b** The fibroblastic iCECs were transformed to polygonal cell shape when exposed to 10 μM SB431542. Scale bar: 100 μm. Immunofluorescence staining showed that the SB431542 treatment rescued Na^+^ K^+^ ATPase expression and reduced fibrosis marker α-SMA. Scale bar: 50 μm

**Supplemental Tables**

**Table. S1 Quality control and in-process tests for the manufacturing of hESC-derived CECs**

| **Target** | **Test** | **Method** | **Release Criteria** | **Test Result** | **Pass or Fail?** |
| --- | --- | --- | --- | --- | --- |
| hESCs | Pluripotent markers | FACS | SSEA-4 ≥ 90% | SSEA-4: 95.9% | PASS |
|  | Sterility test | Rapid sterility testing method | No bacterial or fungal growth | No bacterial or fungal growth | PASS |
|  | Mycoplasma | ELISA method | Negative | Negative | PASS |
| NCCs | NCCs markers | IF | AP2α ≥ 80% | AP2α: 85.30% | PASS |
|  |  |  | SOX10 ≥ 80% | SOX10: 87.25% |  |
|  |  | FACS | P75 ≥ 90% | P75: 95.2% |  |
|  |  |  | HNK1 ≥ 90% | HNK1: 99.7% |  |
|  |  |  | NESTIN ≥ 80% | NESTIN: 98.6% |  |
|  |  |  | SOX2 ≥ 90% | SOX2: 98.9% |  |
|  | Viability | AO/PI staining | Cell count ≥ 80% | Cell count ≥ 90% | PASS |
|  | Sterility | Rapid sterility testing method | No bacterial or fungal growth | No bacterial or fungal growth | PASS |
|  | Mycoplasma | ELISA method | Negative | Negative | PASS |
| CECs | CEC markers | IF | AQP1 ≥ 60% | AQP1: 68.7% | PASS |
|  |  | FACS | ZO-1/ Na^+^ K^+^ ATPase ≥ 90% | ZO-1/ Na^+^ K^+^ ATPase: 96.4% |  |
|  |  |  | N-Cadherin ≥ 90% | N-Cadherin: 97.5% |  |
|  |  |  | CD166 ≥ 90% | CD166: 96.6% |  |
|  | Impurities makers | IF | KI67≤ 1% | KI67≤ 0.1% | PASS |
|  | Undifferentiation markers (*OCT4*) | qPCR method | Cq > 30 | Cq: 33.86 | PASS |
|  | Morphology | Microscopic observation | Cobblestone or hexagonal morphology can be observed | Cobblestone or hexagonal morphology can be observed | PASS |
|  | Viability | AO/PI staining | Cell count ≥ 80% | Cell count ≥ 80% | PASS |
|  | Sterility | Rapid sterility testing method | No bacterial or fungal growth | No bacterial or fungal growth | PASS |
|  | Mycoplasma | ELISA method | Negative | Negative | PASS |
|  | Endotoxin | LAL^b^ turbidimetry test | ≤ 10 EU/ml | < 1 EU/ml | PASS |

**Table. S2 Primers used for qRT-PCR**

| **Gene** | **Direction** | **Sequences** |
| --- | --- | --- |
| *POU5F1* | Forward | 5’ AAA CGA CCA TCT GCC GCT TTG A 3’ |
|  | Reverse | 5’ GGT TGC CTC TCA CTC GGT TCT C 3’ |
| *NANOG* | Forward | 5’ CAG CCC AGA TTC TTC CAC CAG TCC C 3’ |
|  | Reverse | 5’ CGG AAG CTT CCC AGT CGG GTT CAC C 3’ |
| *NGFR* | Forward | 5’ CCT CAT CCC TGT CTA TTG CTC C 3’ |
|  | Reverse | 5’ GTT GGC TCC TTG CTT GTT CTG C 3’ |
| *B3GAT1* | Forward | 5’ GAA AGC AGC CTC CTT CGA GAA C 3’ |
|  | Reverse | 5’ CCT CAT TCA CCA GCA CTG GCT T 3’ |
| *NESTIN* | Forward | 5’ TCA AGA TGT CCC TCA GCC TGG A 3’ |
|  | Reverse | 5’ AAG CTG AGG GAA GTC TTG GAG C 3’ |
| *TFAP2A* | Forward | 5’ GAC CTC TCG ATC CAC TCC TTA C 3’ |
|  | Reverse | 5’ GAG ACG GCA TTG CTG TTG GAC T 3’ |
| *SOX10* | Forward | 5’ ATG AAC GCC TTC ATG GTG TGG G 3’ |
|  | Reverse | 5’ CGC TTG TCA CTT TCG TTC AGC AG 3’ |
| *TJP1* | Forward | 5’ AGT AAG AGC ACA GCA ATG GAG 3’ |
|  | Reverse | 5’ TCA CTA TTG ACG TTT CCC CAC 3’ |
| *CDH2* | Forward | 5’ CCC AAG ACA AAG AGA CCC AG 3’ |
|  | Reverse | 5’ GCC ACT GTG CTT ACT GAA TTG 3’ |
| *AQP1* | Forward | 5’ TGG CTG TGG GAT TAA CCC TG 3’ |
|  | Reverse | 5’ GGT TGC TGA AGT TGT GTG TGA TC 3’ |
| *GAPDH* | Forward | 5’ TTG AGG TCA ATG AAG GGG TC 3’ |
|  | Reverse | 5’ GAA GGT GAA GGT CGG AGT CA 3’ |

**Table. S3 List of antibodies used in FACS.**

| **Antigen** | **Label** | **Dilution** | **Company** | **Cat. No.** |
| --- | --- | --- | --- | --- |
| Anti-human CD271 | PE | 20 µl / test | BD Pharmingen | 560927 |
| Anti-human HNK1 | PE | 5 µl / test | BD Pharmingen | 560844 |
| Anti-human NESTIN | PE | 5 µl / test | BD Pharmingen | 561230 |
| Anti-human SOX2 | PE | 5 µl / test | BD Pharmingen | 562195 |
| Anti-human CD90 | PE | 5 µl / test | Invitrogen | 12-0909-42 |
| Anti-human CD105 | PE | 5 µl / test | Biolegend | 800504 |
| Anti-human CD73 | PE | 5 µl / test | Invitrogen | 12-0739-42 |
| Anti-human CD29 | PE | 5 µl / test | Biolegend | 303004 |
| Anti-human CD19 | PE | 20 µl / test | BD Pharmingen | 555413 |
| Anti-human CD34 | PE | 20 µl / test | BD Pharmingen | 555822 |
| Mouse IgG1 Isotype Control | PE | 20 µl / test | BD Pharmingen | 555749 |
| Anti-human ZO-1 | / | 1:50 | Thermo Fisher Scientific | 61-7300 |
| Anti-Human  Na^+^ K^+^ ATPase | / | 1:50 | EMD Millipore | 05-369 |
| Anti-Human N-cadherin | / | 1:50 | Santa Cruz | Sc-59987 |
| Anti- Human CD166 | PE | 20 µl / test | BD Pharmingen | 559263 |

**Table. S4 Antibodies used in Immunofluorescence Staining**

| **Antigen** | **Host** | **Dilution** | **Company** | **Cat. No.** |
| --- | --- | --- | --- | --- |
| P75 | mouse | 1:100 | ATS | AB-N07 |
| NESTIN | rabbit | 1:1000 | EMD Millipore | ABD69 |
| SOX10 | rabbit | 1:500 | abcam | ab108408 |
| AP2α | mouse | 1:200 | DSHB | 3B5 |
| TUJ1 | rabbit | 1:1000 | Biolegend | 802001 |
| Peripherin | mouse | 1:100 | Santa Cruz | sc-377093 |
| S100β | rabbit | 1:100 | abcam | ab52642 |
| ZO-1 | rabbit | 1:250 | Thermo Fisher Scientific | 61-7300 |
| Na^+^ K^+^ ATPase | rabbit | 1:500 | abcam | ab76020 |
| AQP1 | rabbit | 1:200 | abcam | ab168387 |
| N-cadherin | mouse | 1:100 | Santa Cruz | sc-59987 |
| OCT4 | rabbit | 1:200 | Santa Cruz | sc-9081 |
| KI67 | rabbit | 1:200 | Thermo Fisher Scientific | 710229 |
| STEM121 | mouse | 1:200 | Clontech | Y40410 |
| α-SMA | mouse | 1:500 | Sigma | F3777 |
| Donkey anti-mouse IgG Alexa 488 | Donkey | 1:200 | Jackson ImmunoResearch | 715-545-151 |
| Donkey anti-rabbit IgG Cy^TM^ 3 | Donkey | 1:200 | Jackson ImmunoResearch | 711-165-152 |
| Donkey anti-rabbit IgG Alexa 488 | Donkey | 1:200 | Jackson ImmunoResearch | 711-545-152 |
| Donkey anti-mouse IgG Cy^TM^ 3 | Donkey | 1:200 | Jackson ImmunoResearch | 715-165-151 |
